# Assessment of the applicability of a low-cost sensor–based methane monitoring system for continuous multi-channel sampling

**DOI:** 10.1007/s10661-021-09290-w

**Published:** 2021-07-23

**Authors:** Isura Sumeda Priyadarshana Nagahage, Ekanayaka Achchillage Ayesha Dilrukshi Nagahage, Takeshi Fujino

**Affiliations:** 1 Department of Plant Physiology, Umeå University, 901 87 Umeå, Sweden; 2 Graduate School of Science and Engineering, Saitama University, 255 Shimo-Okubo, Sakura-ku, Saitama, 338-8570 Japan

**Keywords:** Air quality, Methane sensor, Methane monitoring system, Multi-channel sampling

## Abstract

Systems that are made of several low-cost gas sensors with automatic gas sampling may have the potential to serve as reliable fast methane analyzers. However, there is a lack of reports about such types of systems evaluated under field conditions. Here, we developed a continuous methane monitoring system with automated gas sampling unit using low-cost gas sensors, TGS 2611 and MQ-4, that use a simple cloud-based data acquisition platform. We verified the consistency, repeatability, and reproducibility of the data obtained by TGS 2611 and MQ-4 low-cost gas sensors by measuring high- and low-concentration methane samples. The normalized root-mean-square errors (NRMSEs) of the samples with high methane concentrations, [CH_4_] of 3, 4, 6, and 7%, were 0.0788, 0.0696, 0.1198, and 0.0719 for the TGS 2611 sensor, respectively, and were confirmed using a gas chromatograph as a reference analyzer. The NRMSEs of the samples with low [CH_4_] of 0.096, 0.145, 0.193, and 0.241% measured by the TGS 2611 sensor were 0.0641, 0.1749, 0.0157, and 0.1613, whereas those NRMSEs of the same concentrations measured by the MQ-4 sensor were 0.3143, 0.5766, 0.6301, and 0.6859, respectively. Laboratory-scale anaerobic digesters were tested using the developed system. The anaerobic digesters were continuously operated for 2 months, demonstrating the potential use of sensors for detecting and monitoring methane in the field level application. This study utilized a unique way to combine the advantages of low-cost sensors and develop a reliable monitoring system by minimizing drawbacks of low-cost sensors.

**Supplementary Information:**

The online version contains supplementary material available at 10.1007/s10661-021-09290-w.

## Introduction

Air quality monitoring has become a critical requirement due to the current rise in health issues associated with air pollution, climate change, and impaired quality of life (Bentayeb et al., 2015; Manisalidis et al., 2020; Pascal et al., 2013; Raaschou-Nielsen et al., 2016; Wu et al., 2016). Therefore, many research works have been carried out in this research area to develop new technologies, evaluation of these techniques, and application (Benaissa et al., 2019; Eugster et al., 2020; Gibergans-Baguena et al., 2020; Wu et al., 2019).

With the concern about the direct impact of methane (CH_4_) on climate change and human health (Isaksen et al., 2014), it is important to continuously monitor atmospheric methane spatially and temporally.

Gas fluxes from different sources can be measured using measurement approaches such as the point-scale chamber technique as well as micrometeorological techniques (National Academies of Sciences, Engineering, and Medicine, 2018). Gas samples can be detected using various analyzers, including gas chromatography-flame ionization detectors (GC-FID), Fourier transform infrared (FTIR) spectroscopic methods and optical gas imaging (Oliver, 2019). These measurement techniques require expensive instruments and technical knowledge to operate them, and the measurements are carried out intermittently owing to the cost of the analysis. Although many detection and measurement technologies exist, each method has its limitations and only a few available technologies have real-time, continuous monitoring capabilities (Hu et al., 2014).

Thus, some researchers have investigated the potential use of low-cost gas sensors for atmospheric methane monitoring in laboratory-level and field-scale implementations (van den Bossche et al., 2016; Spinelle et al., 2017; Collier-Oxandale et al., 2018; Yang et al., 2019). Also emerging advanced solid-state sensing devices (artificial olfaction by e-noses) that employ sensors and algorithms based on artificial neural networks have been developed (Hu et al., 2018). Moreover, low-cost sensors have attracted the interest of researchers not only because of their low cost but also because of other desirable features such as their smaller size, lower weight, and reduced power consumption as well as their same analytical principles as established reference instruments (Lewis et al., 2018). Interestingly, these low-cost sensors are capable of detecting and measuring atmospheric compositions, focusing on reactive air pollutants (CO, NOx, O_3_, and SO_2_), particulate matter (PM), and the greenhouse gases CO_2_ and CH_4_ (Lewis et al., 2018).

Thus, applicability of low-cost sensors provides an insight into different implementations and developments of sensor-based systems.

However, low-cost sensors have several limitations when it comes to the application. The main drawbacks of low-cost methane sensors are that their low accuracy, limited measurement range, sensor-to-sensor variability, and durability (Honeycutt et al., 2019). In addition, it is known that these sensors’ accuracy depends on environmental parameters such as air temperature and humidity (Eugster & Kling, 2012). Therefore, applications of these sensors are limited to tasks where precise measurements are not required.

But, there could be a possibility to improve above limitations by combining several low-cost sensors, and utilize same sensor system to measure different samples automatically. However, there is a lack of studies that examine low-cost methane monitoring systems which are made of several low-cost sensors and having the ability to measure multiple samples by using the same monitoring system.

The objective of the present study was to develop a low-cost continuous methane monitoring system and minimize limitations of low-cost sensors by combining two low-cost methane sensors to measure multiple samples from different sources. The developed system was consisted with TGS 2611 and MQ4 sensors that have been evaluated for accuracy and reliability under laboratory conditions independently (Eugster & Kling, 2012; van den Bossche et al., 2016; Honeycutt et al., 2019). In addition, we combined temperature and humidity sensor to study the effect of air temperature and the humidity of the sample on methane measurement. We employed a high-dilution technique to facilitate methane detection by low-cost sensors in their operating detection range. Then, the developed system was coupled with the anaerobic digesters to facilitate the continuous detection of low-concentration methane. Our study suggested that combination of MQ4 and TGS 2611 sensors have improved range of detection, accuracy, and decreased sensor-to-sensor variability.

## Materials and methods

### Selected low-cost sensors for methane monitoring system

A TGS 2611-metal–oxide–semiconductor (MOS)-type gas sensor (Figaro Engineering Inc., Osaka, Japan) (TGS-2611 sensor technical data, 2019) was used as the low-cost gas sensor to detect the methane concentrations of the anaerobic digesters. The typical detection range of the sensor is 500–10,000 ppm, the circuit voltage of the sensor is 5 V, and the sensing material of the sensor is SnO_2_. Suitability of sensor-based atmospheric methane monitoring was previously evaluated (Eugster & Kling, 2012; van den Bossche et al., 2016; Honeycutt et al., 2019).

MQ-4 is a semiconductor-type gas sensor (Hanwei Electronics Group Corporation, Zhengzhou, China), with a high sensitivity to methane and a circuit voltage of 5 V. The sensor is capable of detecting methane gas in the concentration range of 200–10,000 ppm. This sensor is composed of a micro size Al_2_O_3_ ceramic tube, a SnO_2_ sensitive layer, a measuring electrode, and a heater fixed in a layer consisting of a plastic and stainless steel net (MQ-4 sensor technical data, 2019). MQ-4 sensor has been studied in low-cost application on biogas measurement by Yang et al. (2019). Moreover, MQ sensors are capable of detecting different hydrocarbons, which have been used in accordance with their specifications.

The operating principle of semiconductor-type sensors is based on changes in resistance as a result of changes in adsorbed oxygen concentration (Lee et al., 2018). In clean air, donor electrons on a semiconductor surface are attracted towards oxygen, reducing the flow of electric current. In the presence of reducing gases, the amount of adsorbed oxygen is decreased through the release of electrons into the semiconductor material, allowing current to flow (Figaro Engineering Inc.). Furthermore, this type of sensor requires a minimum conditioning period or preheating time. The preheating facilitates the heating element and allows the sensing element of the sensor to be consistently heated (TGS 2611: 7 days of conditioning before the start of the test and MQ-4 sensor: preheating for 24 h).

### Automated methane monitoring system for the detection of methane concentration

**Fig. 1:**
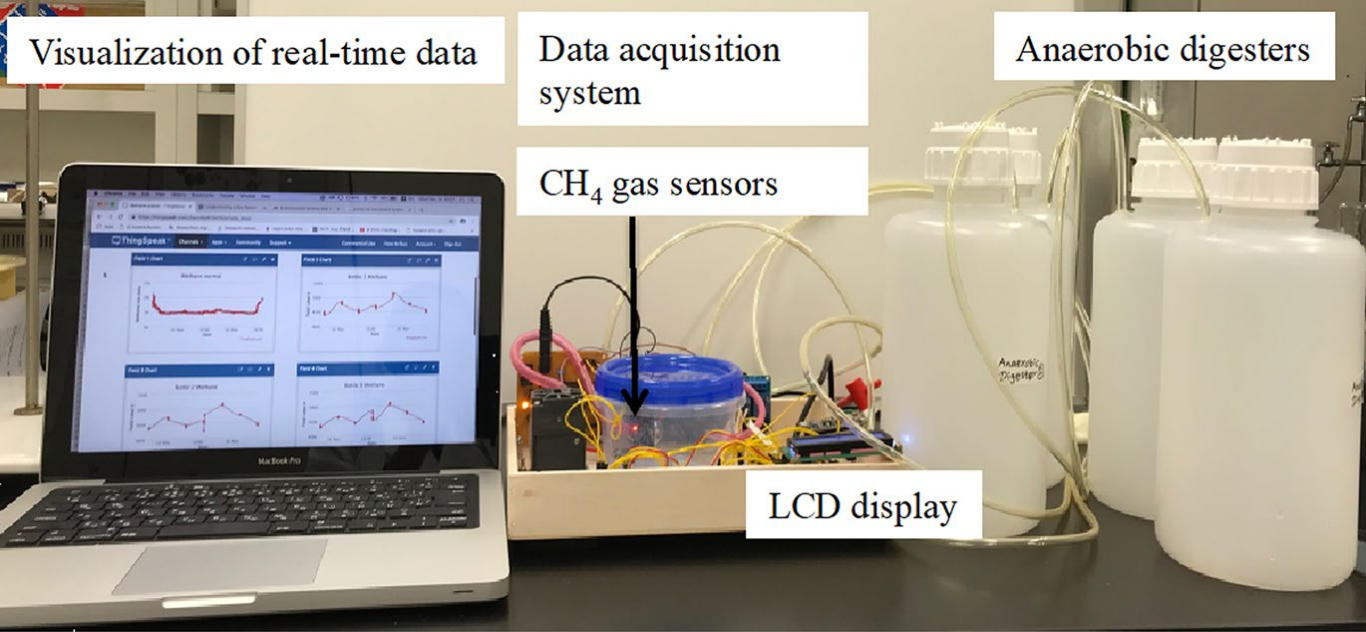
Acquisition and visualization of real-time data by the methane monitoring system under clean-air conditions

**Fig. 2:**
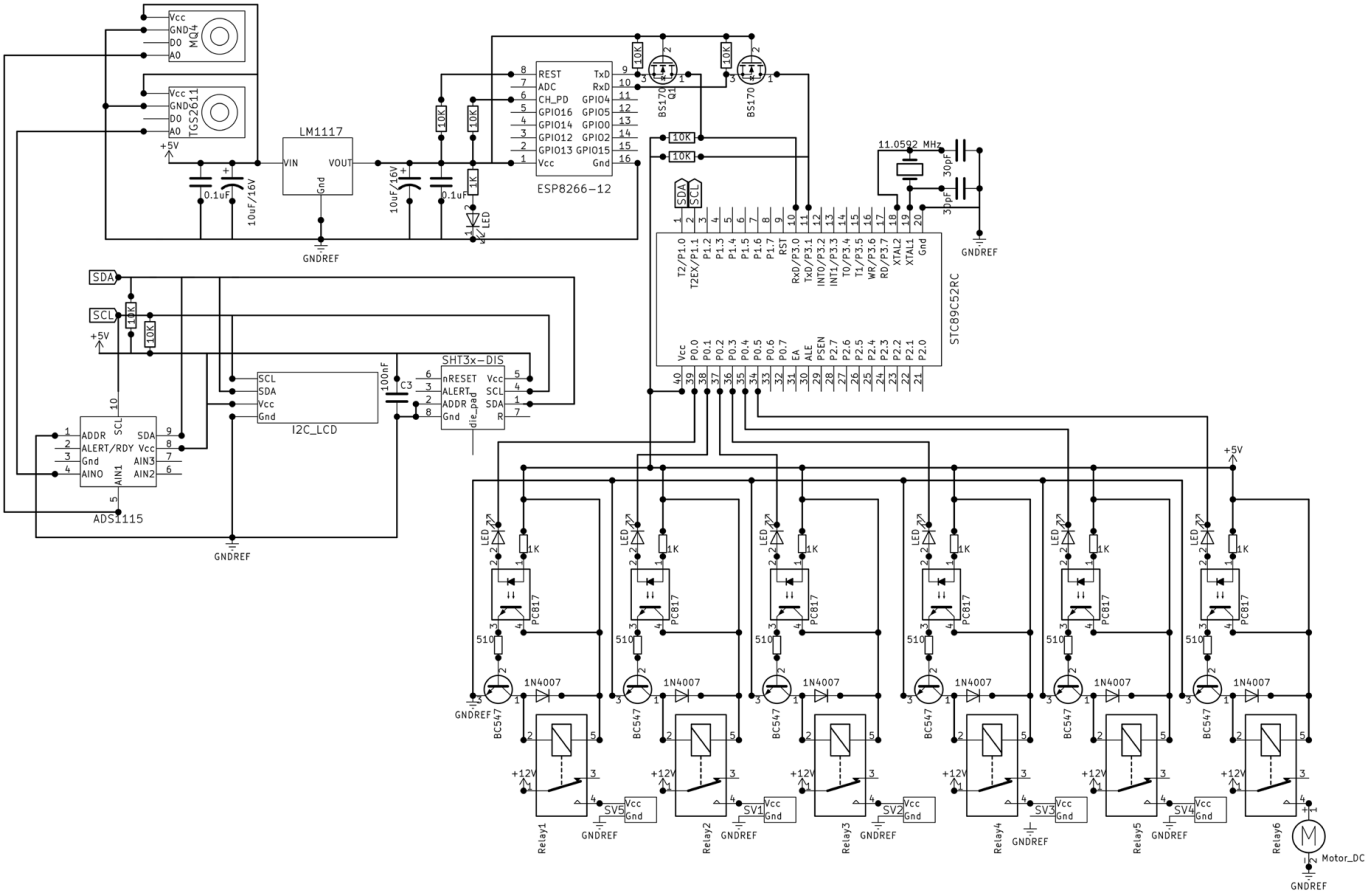
Circuit diagram of the methane monitoring system

The automated methane monitoring system mainly performed the automated sampling, detection, and monitoring of methane gas, followed by data acquisition and storage (Figs. 1, 2).

### Sensor arrangement in vacuum chamber

**Fig. 3:**
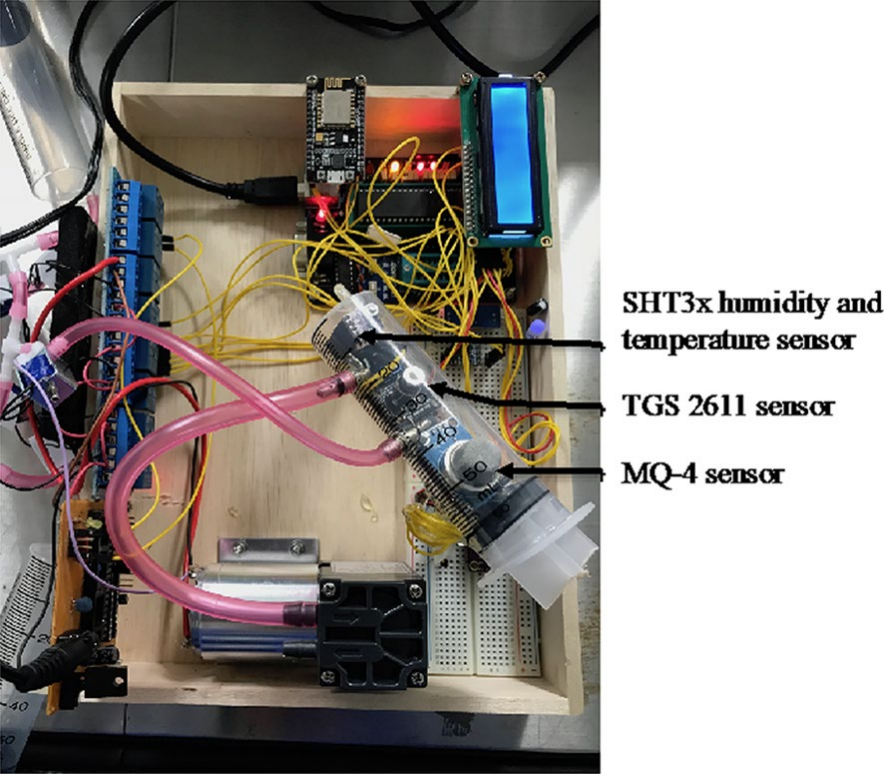
Sensor arrangement inside the vacuumed syringe chamber

The TGS 2611 and MQ-4 sensors and an SHT3x humidity and temperature sensor were arranged in a 50-mL syringe to obtain an air sample with a fixed volume for measurement (Fig. 3). The inlet and outlet of the 50-mL syringe (hereafter known as vacuum chamber) were connected via a one-way valve to secure the air sample.

During the measurement, seven readings per sample were obtained during one measuring cycle. The vacuum chamber was flushed with clean air for 3 min before the start of the next measuring cycle. The temperature and humidity of the air sample and the atmosphere were obtained by the SHT3x humidity and temperature sensor.

### Instrumentation for gas sampling

**Fig. 4:**
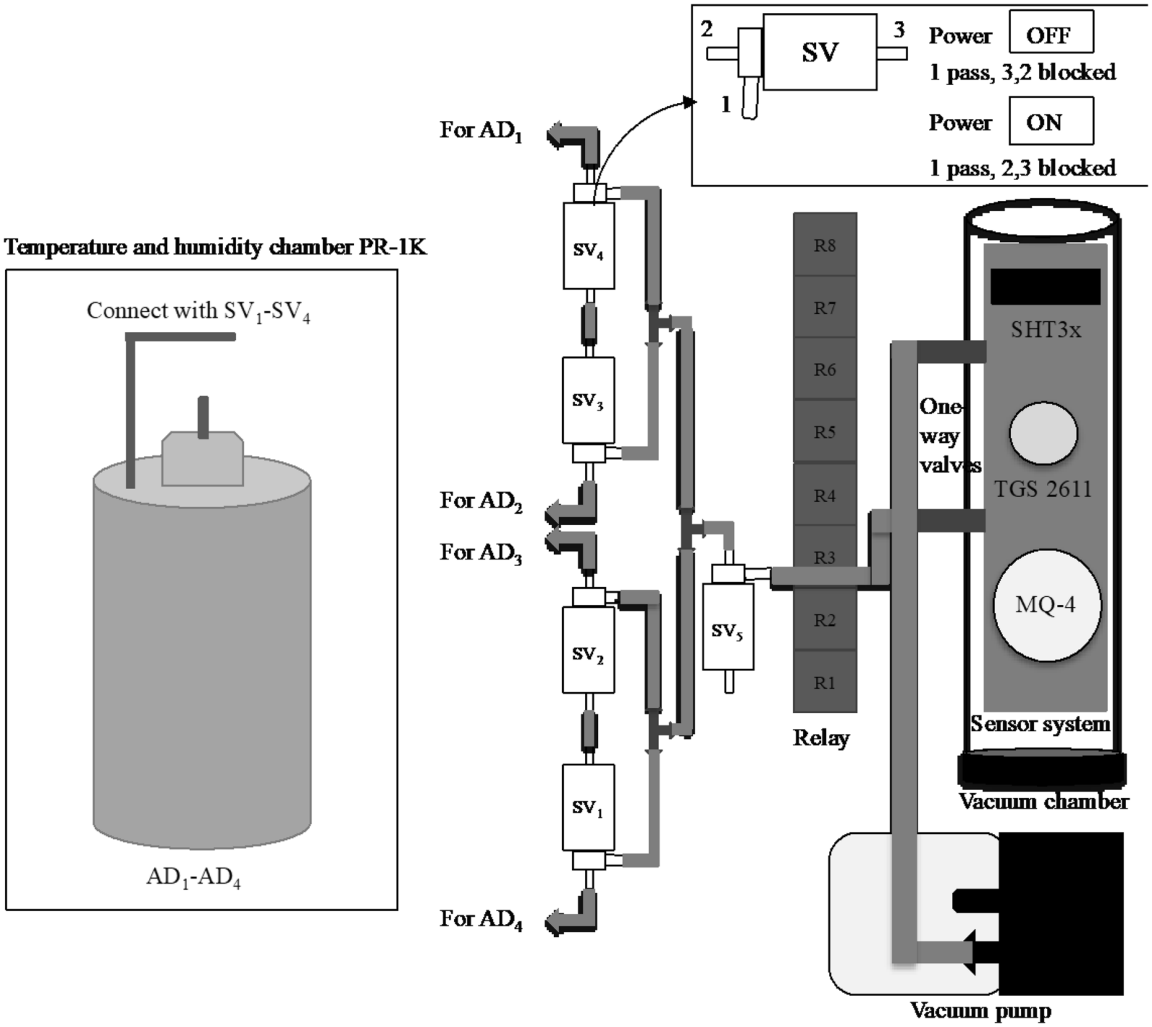
Sensor and relay arrangement of multi-channel sampling unit

Anaerobic digesters (AD_1_–AD_4_) were connected with the vacuum chamber via three-way solenoid valves (SV_1_–SV_5_). The gas samples obtained from AD_1_ to AD_4_ passed through the SV_1_–SV_4_, respectively, using SV_5_ as the common entry to the vacuum chamber (Fig. 4). The gas sampling from each anaerobic digester was carried out separately and included a flushing cycle with clean air after each measurement. The solenoid valves were operated by a relay board connected to the main microcontroller (STC89C52RC). During each measurement, the air sample entered the vacuum chamber owing to the vacuum generated by the vacuum pump (DC 12 V, 6 W mini vacuum pump). The common three-way solenoid SV_5_ was connected to the vacuum chamber via a one-way valve. Similarly, the flushed air was removed from the vacuum chamber via another one-way valve to prevent the gas from mixing with other gas samples during the measurements.

### Instrumentation for data acquisition

The data acquisition system was similar to that in our previous study (Nagahage et al., 2019). It consisted of two main components: a microcontroller unit and a WiFi module. The main microcontroller (STC89C52RC) was operated at a speed of 11.0592 MHz. A software-implemented *I*^2^C bus was used to interface a 16 × 2 LCD module, an ADS1115 16-bit analog-to-digital converter (ADC), and a temperature and humidity sensor with the microcontroller. An ESP8266-12E low-cost serial-to-WiFi module was interfaced through STC89C52RC inbuilt UART. The analog data output pins of the TGS 2611 and MQ-4 gas sensors were connected to the ADS1115 with a full-scale range of ± 4.096 V. *ThingSpeak* API, an open IoT (Internet of Things) platform, was used to collect and analyze data with MATLAB^@^ analytics. The assembly program (supplemental material 1) for the microcontroller was written using Keil µVision 5 IDE, and AT commands were used to control the WiFi module (Figs. 1, 2). The total cost of the developed prototype was $84.31 (Table 1).

**Table 1:** Total cost of the developed methane monitoring system (US$ in 2019)

Component	Units	Unit cost ($)	Subtotal ($)	Total ($)
Methane monitoring and data acquisition				
STC89C52RC	1	1.12	1.12	
ADS1115	1	2.73	2.73	
ESP8266-12E	1	1.79	1.79	
TGS 2611	1	35.84	35.84	
MQ-4	1	1.29	1.29	
SHT30	1	3.98	3.98	
LCD1602	1	2.12	2.12	
Components for gas sampling				
Three-way solenoid valves	5	2.55	12.75	
Vacuum pump (DC 12 V 6 W)	1	15.70	15.70	
Relay board	1	1.99	1.99	
Other components			5.00	
				84.31

### Implementation of anaerobic digesters for data acquisition

Anaerobic digesters with four different conditions/treatments were used for continuous methane monitoring. The digesters were operated for 2 months (hydraulic retention time, (HRT) of 60 days) with the continuous monitoring of the methane production.

Anaerobic digesters were constructed using 2-L heavy-duty vacuum bottles filled with 400 mL of palm oil mill effluent (POME) and 5.0 g of activated sludge to start the digestion (Hamzah et al., 2019). The POME was obtained from a palm oil processing factory in Malaysia. The effluent was stored at 4 °C in a refrigerator prior to the experiment. The anaerobic digesters were maintained under a mesophilic condition (37 °C) inside a PR-1 K temperature and humidity chamber (Espec Tabai Corp. Japan).

### Conversion of raw data into methane concentration in ppm

The methane concentrations of anaerobic digesters were obtained using low-cost sensors. The raw data of the sensors were stored in the *ThingSpeak* platform. We performed raw data conversion using the manufacturer’s calibration graphs (see the calibration graphs for clean air and methane in the manufacturer’s data sheet for the TGS 2611 gas sensor and MQ-4 gas sensor) to obtain the methane concentration in ppm. The data conversion using the accurately calibrated manufacturer’s function obtained from the graphs (TGS 2611 and MQ-4 technical data) was performed satisfactorily, and we confirmed the data with a reference analysis using a gas chromatograph (GC-2014, Shimadzu, Japan).

The methane concentration in ppm was calculated using the following mathematical relationships (TGS 2611 and MQ-4 technical data) and the general *V* = *IR* relationship as follows:

1 $${V}_{\mathrm{RL}}=\frac{{V}_{C}}{{2}^{15}}$$

2 $${R}_{S}={R}_{L}\left(\frac{{V}_{C}}{{V}_{\mathrm{RL}}}-1\right)$$

3 $$\left[{\mathrm{CH}}_{4}\right]={10}^{\left\{\left[\left(\frac{\mathrm{log}{R}_{S}}{{R}_{0}}\right)-b\right]/m\right\}}$$

where *V*_RL_ is the voltage at the load resistor (*R*_L_), *V*_C_ is the circuit voltage (5 V), *R*_S_ is the sensor resistance, *R*_0_ is the sensor resistance at a methane concentration of 1000 ppm in clean air, and *b* and *m* are the intercept and slope of the manufacturer’s calibration function, respectively. The predefined value of 2^15^ is the value used for ADC conversion.

### Validation of the sensor reading using a GC as a reference analyzer

Preliminary studies were conducted to evaluate the repeatability and consistency of the measured data obtained from the developed methane monitoring system. Gas samples with methane concentrations of 3, 4, 6, and 7% were prepared using 99.9% standard methane gas. Automated gas sampling was performed using 50 mL syringes that were connected to the tubes of the methane monitoring system. Seven measurements were obtained for each concentration by the methane monitoring system. The same experiment was performed twice to confirm the measurements. The TGS 2611 sensor is capable of detecting of high-concentration methane samples, which was confirmed using a gas chromatograph as a reference analyzer. However, the MQ-4 sensor was unable to detect high-concentration samples since the concentrations exceeded their detection limit, and it produced the same raw sensor response to all the concentrations. Thus, we used a high-dilution method to evaluate the response of the sensors in their detection ranges.

### Preparation of gas samples using high-dilution method

We diluted the methane gas in 2-L-heavy-duty bottles to obtain very low concentrations by the following procedure: We injected 2, 3, 4, and 5 mL of standard methane gas into the 2-L-heavy-duty bottles that were connected to the methane monitoring system via 150-cm long tubes. The prepared low-gas concentrations in the 2 L volumes were 0.096, 0.145, 0.193, and 0.241%, respectively.

### Evaluation of sensor repeatability and consistency

An experiment to evaluate repeatability and consistency was conducted using the 2-L-heavy-duty bottles before the experiment on anaerobic digestion. In this experiment, methane gas was injected into empty 2-L-heavy-duty bottles (labeled AD_1_–AD_4_) with volumes of 2, 3, 4, and 5 mL of 99.9% standard methane gas. The expected methane concentrations of the AD_1_–AD_4_ were 0.097, 0.145, 0.193, and 0.241%, respectively. The length of the gas tube from each 2-L-heavy-duty bottle to the vacuum chamber was considered when calculating the concentration of the methane gas inside the bottle.

### Statistical analysis

To evaluate the repeatability and consistency, preliminary studies were carried out using known standard methane concentrations. We calculated the root-mean-square error (RMSE) as follows to evaluate the methane concentration measured by the gas sensors and gas chromatograph:

4 $$\mathrm{RMSE}=\sqrt{\frac{1}{n}\sum_{i=0}^{n}{(\theta -{\theta }_{p})}^{2}},$$

where *θ* is the methane concentration measured by the gas chromatograph (m^3^ m^−3^) or the methane gas concentration calculated by the high-dilution method, *θ*_*p*_ is the methane concentration measured by the gas sensors (m^3^ m^−3^), and *n* is the number of measurements in each measuring cycle. As a quantitative measure of prediction performance, we calculated the normalized root mean square error (NRMSE) by dividing the RMSE using the mean concentration of observations of the respective sensor (Kuula et al., 2019; Smith et al., 2019).

### Temperature and humidity dependence

The temperature and humidity of the air sample and atmosphere were obtained by an SHT3x humidity and temperature sensor (Nagahage et al., 2019). The conversion of the temperature (result in °C) and humidity (result in %RH) signal output was performed using the following equations (Humidity & Temperature Sensor Datasheet SHT3x-DIS, 2016):

Temperature conversion

5 $$T\left[^\circ \mathrm{C}\right]=-45+175\frac{{S}_{T}}{{2}^{16}-1}$$

Relative humidity conversion

6 $$RH=100\frac{{S}_{RH}}{{2}^{16}-1}.$$

where *S*_*T*_ and *S*_*RH*_ denote the raw sensor output for humidity and temperature, respectively.

The correlation coefficients of the parameters were evaluated using a simple linear regression model. The coefficient values were calculated to obtain the best fit for the parameters.

## Results and discussion

### Automated gas sampling in vacuum chamber for measurements

A gas sample from an anaerobic digester was collected in the vacuum chamber for each measurement. A complete measurement cycle consisted of a 40-min measuring cycle followed by 20 min of atmospheric air flushing with a rest period. The methane monitoring system monitored four anaerobic digesters in a complete measuring cycle. Thus, it took 1 h to complete the measurement of all four anaerobic digesters in one round. The methane concentration of a gas sample from each anaerobic digester was repeatedly measured seven times. The data acquisition system recorded a measurement every minute; thus, it took 7 min to take seven readings from one air sample from an anaerobic digester. Intermittent air flushing was performed between times when gas samples were taken from different digesters (AD_1_–AD_2_, AD_2_–AD_3_, and AD_3_–AD_4_, with complete air flushing and a rest period at the end of the measurements). The intermittent air flushing had a duration of 2–3 min.

### Automated data acquisition

The raw data obtained from the gas sensors and the temperature and humidity sensor were stored in the *ThingSpeak* platform. The methane concentration of the atmospheric air, and the temperature and humidity of the air were recorded repeatedly every minute. Also, the changes in methane concentration, temperature, and humidity in each complete measuring cycle for all anaerobic digesters were recorded once per day. In other words, each anaerobic digester was measured once a day, and one gas sample taken from a bottle was repeatedly measured seven times.

### Data validation using GC as a reference analyzer

Automated gas sampling was performed to inject a gas sample into the vacuumed chamber. Thus, we were able to maintain the same experimental conditions for all the sensors used in the methane monitoring system. NRMSE was calculated for the methane concentration ([CH_4_]) in % measured from the gas chromatograph and TGS 2611 gas sensor. The NRMSEs for the samples with 3, 4, 6, and 7% methane concentrations were 0.0788, 0.0696, 0.1198, and 0.0719, respectively (Table 2).

**Table 2:** RMSE and NRMSE of TGS and MQ4 sensors at high and low methane concentrations

	TGS		MQ-4	
[CH_4_] %	RMSE	NRMSE	RMSE	NRMSE
3	0.2231	0.0788	-	-
4	0.2795	0.0696	-	-
6	0.6380	0.1198	-	-
7	0.4973	0.0719	-	-
0.096	0.0062	0.0641	0.0302	0.3143
0.145	0.0254	0.1749	0.0836	0.5766
0.193	0.0030	0.0157	0.1216	0.6301
0.240	0.0389	0.1613	0.1653	0.6859

In addition, ANOVA was conducted to determine statistical significance between TGS sensor and GC measurements (Table 3). The results showed that there was no statistical significance between TGS 2611 and GC measurements, suggesting that the accuracy of the gas sample concentration measurement by the TGS 2611 sensor is acceptable for gas monitoring purposes.

**Table 3:** ANOVA table to validate the TGS 2611 sensor data with GC. The results indicate no significant difference between the TGS 2611 measurements and GC measurement at 5% significant level

	Df	Mean sum of squares	*F* value	*P* value
GC to TGS	1	0.0780	0.0321	0.8589
residual error	29	2.4284		

However, the MQ-4 sensor was unable to provide acceptable values owing to its detection limitation and showed a limited raw response to subsequent methane concentrations. Even though the maximum detection limit of both sensors is 10,000 ppm, the TGS 2611 sensor was able to perform satisfactorily by changing its raw response to the selected methane gas concentration. Alternative setup could be used to improve measurement range of MQ-4 sensors, as injection of gas into a partially closed capsule facilitated the measurement of high methane concentration (Fakra et al., 2020).

This result suggested that both the TGS 2611 and MQ-4 sensors may perform satisfactorily within the given detection range. On the basis of this result, the repeatability and consistency experiment was performed using the gas samples prepared within the detection range of the gas sensors.

### Sensor repeatability and consistency

**Fig. 5:**
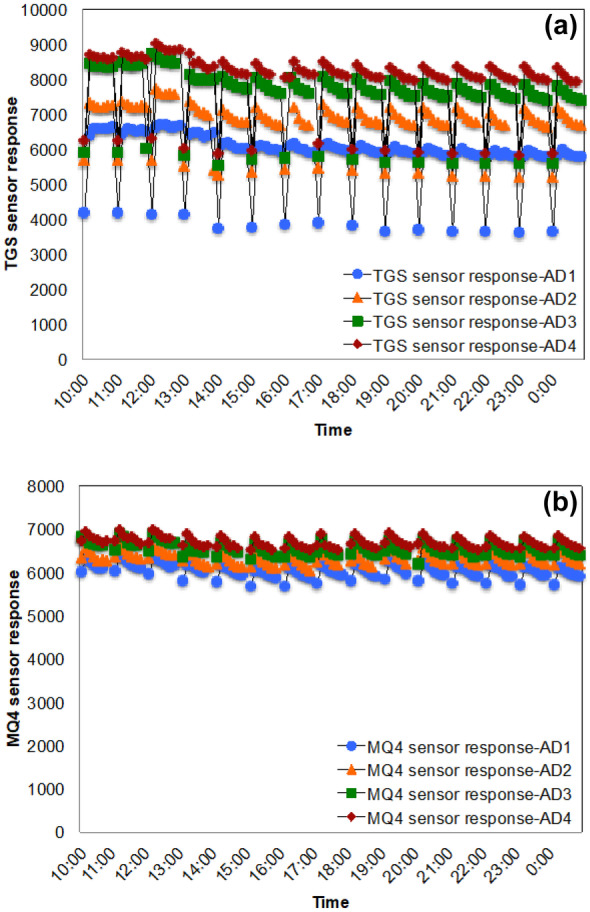
Change in gas sensor response with time during the repeatability and consistency experiment: (**a**) TGS 2611 sensor response for AD_1_–AD_4_, (**b**) MQ-4 sensor response for AD_1_–AD_4_ (Note that during the 1 h complete measurement cycle, the measurements from AD_1_ to AD_4_ were configured/recorded as consecutive measurements.) In the figure, the readings of the four bottles are shown within the same time period (not as consecutive measurements for this time period) to increase the readability

The sensor repeatability and consistency were evaluated for both sensors using low methane concentrations. The different concentrations of methane in the 2-L heavy-duty bottles were measured repeatedly for 15 h. The methane monitoring system connected to the data acquisition system recorded all the measurements of the 2-L heavy-duty bottles labeled AD_1_–AD_4_ (Fig. 5a, b). The response time of the TGS 2611 gas sensor was faster than that of the MQ-4 gas sensor as it responded to atmospheric [CH_4_] (a response of ~ 4000) during each intermittent air flushing. However, the MQ-4 sensor was not capable of detecting atmospheric methane concentration during each intermittent air flushing and started to respond to the next gas sample. Honeycutt et al. (2019) also observed higher settling time of MQ-4 sensor than TGS 2611 sensor.

The results suggest that the MQ-4 sensor requires a longer response time to perform satisfactorily.

In addition, the accuracy of both sensors at low methane concentrations was studied by calculating NRMSEs (Table 2). The calculated NRMSEs of the [CH_4_] of 0.096, 0.145, 0.193, and 0.241% measured by the TGS 2611 sensor were 0.0641, 0.1749, 0.0157, and 0.1613, whereas those NRMSEs of the same concentrations measured by the MQ-4 sensor were 0.3143, 0.5766, 0.6301, and 0.6859, respectively. The experimental results demonstrate satisfactory performance of both sensors while better accuracy of TGS 2611 sensor over MQ-4 sensor in low methane concentrations.

### Temperature and humidity dependence

**Fig. 6:**
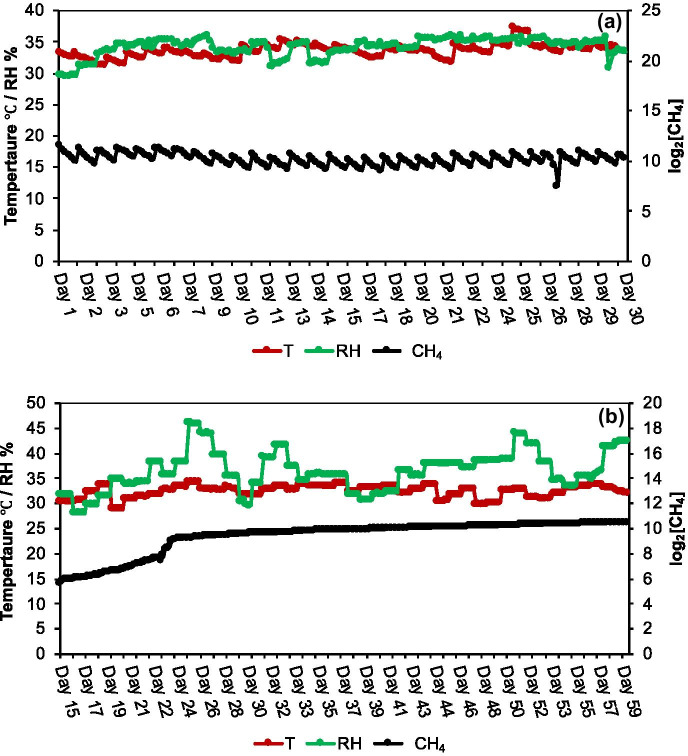
Changes in temperature and relative humidity during the gas space measurement: (**a**) using a known concentration of CH_4_ in AD_3_ before the experiment, (**b**) CH_4_ in AD_4_ during the experiment

**Fig. 7:**
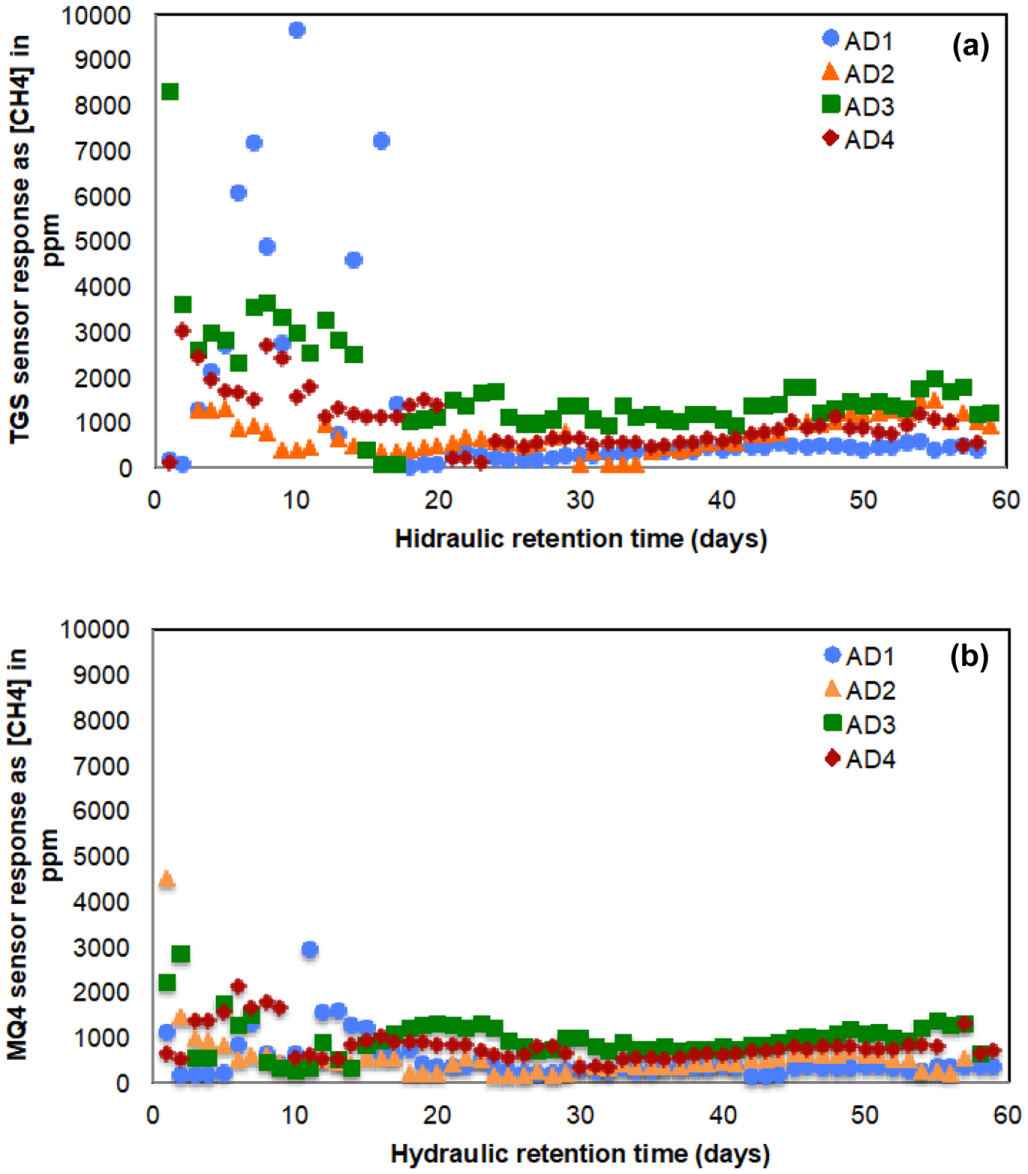
Changes in sensor response as [CH_4_] in ppm during HRT of 60 days for four anaerobic digesters: (**a**) TGS 2611 sensor response as [CH_4_] in ppm, (**b**) MQ-4 sensor response as [CH_4_] in ppm for HRT of 60 days

As mentioned by manufacturers of TGS 2611 and MQ-4 sensors and earlier studies (Eugster & Kling, 2012), these sensors are sensitive to relative humidity and temperature. The temperature and humidity data obtained for fixed amount of [CH_4_] in AD_3_ before anaerobic digestion experiment (Fig. 6a) and the measurement of [CH_4_] in AD_4_ during the anaerobic digestion experiment were selected to determine the temperature and humidity dependence of the sensor response (Fig. 6b). We considered the data after an HRT of 15 days to avoid the initial fluctuation of the data during the establishment of the anaerobic digesters (see Fig. 7). The change in methane concentration for the anaerobic digesters gradually increased after the establishment of the system. Thus, we used this stage to evaluate the temperature and humidity dependence of the sensor reading.

The variations in temperature and the humidity were very limited owing to the controlled environmental conditions inside the vacuum chamber before anaerobic digestion (temperature 29 to 36 °C, humidity 29 to 36%) and during anaerobic digestion (temperature 28 to 34 °C, humidity 28 to 46%) experiments (Fig. 6a, b). The vacuum chamber was fixed on the top of the PR-1 K temperature and humidity chamber. The temperature of the air sample slightly changed during the sampling of air space gases from the anaerobic digesters. Furthermore, the relative humidity of the air sample was low owing to the larger gas space inside the anaerobic digester and may be due to the low evaporation rate of POME.

The dependence of the response on the temperature and humidity during anaerobic digestion was evaluated by regression analysis. The sensor response did not depend on the temperature or humidity of the air sample in our study (*R*^2^ = 0.02 and 0.16, respectively).

### Sensor measurements for laboratory-scale anaerobic digesters

Four anaerobic digesters were operated as the laboratory implementation to evaluate the performance of the methane monitoring system. The anaerobic digesters were operated for an HRT of 60 days with different treatments. The change in methane concentration was monitored using the methane gas monitoring system then recorded (Fig. 7a, b). The data fluctuation was higher in the TGS 2611 sensor response than in the MQ-4 sensor response during the initial 15 days. According to Fig. 7a, b, the TGS 2611 sensor recorded more outliers than the MQ-4 sensor response, which was more stable during our experiment. After HRT of 15 days, both sensor responses were stable for the remainder of the experiment, showing the potential use of these low-cost sensors for atmospheric monitoring systems. Izumoto et al. (2018) demonstrated the importance of evaluation of methane emission at a time scale in landfill sites. However, methane emission varies between different layers of the landfill site (Pehme et al., 2020). Since our system can be utilized to measure methane emission through multiple channels, it will be worthwhile to investigate the applicability of the system in landfill sites to study methane emission more accurately.

### Relationship between measured methane concentration and sensor response

**Fig. 8:**
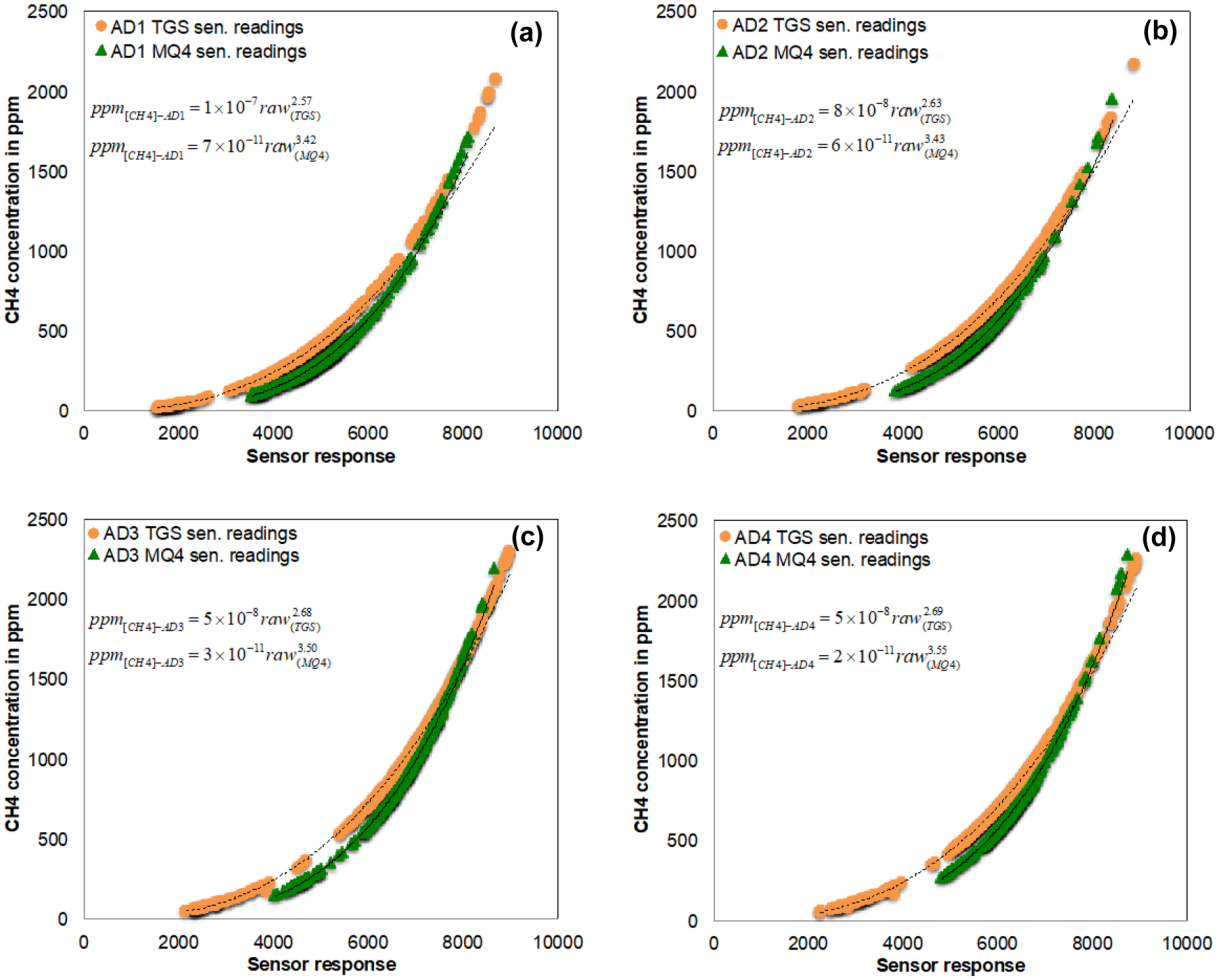
Relationship between the measured [CH_4_] and responses of TGS 2611 and MQ-4 gas sensors: (**a**–**d**) anaerobic digesters AD_1_ to AD_4_

Mathematical relationships between the measured methane concentrations and the responses of the TGS 2611 and MQ-4 gas sensors were derived for the anaerobic digesters (Fig. 8). The outliers of the methane concentrations measured by the sensors were removed, and only the stabilized data were used. To test the hypothesis that the TGS 2611 and MQ-4 sensor readings for the anaerobic digester [CH_4_] in ppm was equal, an ANOVA test was performed. The results of the ANOVA test showed statistically significant differences between the values in ppm measured by both sensors (*P* < 0.05).

Even though their responses are significantly different, the trend of the mathematical relationship (power function) is similar for the two sensors (Fig. 8). Thus, we performed Spearman’s correlation analyses between TGS 2611 sensor and MQ-4 sensor by using methane concentration and sensor raw values. According to the analysis, there was a significant positive correlation in methane concentration values (rho = 0.9520 at *P* = 2 × 10^−16^) and sensor raw values (rho = 0.9542 at *P* = 2 × 10^−16^). The inaccuracy of sensors can be minimized by the development of statistical methods to correct measurements from low-cost sensors (Considine et al., 2021). Therefore, the following regression models were developed to explain the relationship between the two sensors (Table 4). These linear regression models will be utilized in future studies for sensor correction algorithms, cross-validation, and predicting sensor values when one sensor is failed.

**Table 4:** Summary of the regression models. All regression models are statistically significant

	Estimated coefficients	*P* value	Adjusted *R*^2^
General linear model coefficients for [CH4] in ppm			
Intercept	− 6.5902 × 10^2^	2 × 10^−16^	0.3399
MQ-4	2.7550	2 × 10^−16^	
Intercept	4.685 × 10^2^	2 × 10^−16^	0.3399
TGS	1.235 × 10^−1^	2 × 10^−16^	
General linear model coefficients for methane sensor raw values			
Intercept	− 6.2591 × 10^3^	2 × 10^−16^	0.7965
MQ-4	2.0893	2 × 10^−16^	
Intercept	3.584 × 10^3^	2 × 10^−16^	0.7965
TGS	3.813 × 10^−1^	2 × 10^−16^	

## Conclusions

We developed a low-cost gas sensor system for monitoring methane concentration that uses a simple cloud-based data acquisition platform. We tested the performance of the low-cost methane monitoring system by combining it with anaerobic digesters. The TGS 2611 sensor showed a higher response to changes in methane gas concentration. Moreover, the MQ-4 sensor was capable of detecting methane gas within its detection range. However, the TGS 2611 sensor was capable of accurately detecting methane gas above its detection range. In this study we employed the high-dilution method to prevent incorrect gas detection when the concentration exceeded the detection limit. Importantly, we performed a gas sampling procedure involving automated gas sampling in the vacuum chamber for measurements. The strategies we used for gas sampling may be useful for enhancing the durability of sensors and the accuracy of measurements. However, further studies are required to evaluate the sensitivity of both sensors on temperature and humidity. Additional investigations should be performed to check how stable the calibration functions over the time and accuracy of the function in mixed air. This study utilized a unique way to combine the advantages of sensors, circuit control, and data analysis so as to realize a low-cost but reliable monitoring system by minimizing drawbacks of low-cost sensors.

## Supplementary Information

Below is the link to the electronic supplementary material.

Supplementary file1 (PDF 53 KB)

## Data Availability

Some or all data, models that support the findings of this study are available from the corresponding author upon reasonable request.

## Code availability

The codes of the automated data acquisition system are available from the corresponding author upon reasonable request.

## References

[CR1] Benaissa, F., Bendahmane, I., Bourfis, N., Aoulaiche, O., & Alkama, R. (2019). Bioindication of urban air polycyclic aromatic hydrocarbons using Petunia hybrida. *Civil Engineering Journal*, 4 (2), 1305–1313. 10.28991/cej-2019-03091333

[CR2] Bentayeb M, Wagner V, Stempfelet M, Zins M, Goldberg M, Pascal M, Larrieu S, Beaudeau P, Cassadou S, Eilstein D, Filleul L, Tertre A, Medina S, Pascal L, Prouvost H, Quénel P, Zeghnoun A, Lefranc A (2015). Association between long-term exposure to air pollution and mortality in France: A 25-year follow-up study. Environmental International.

[CR3] Collier-Oxandale A, Casey JG, Piedrahita R, Ortega J, Halliday H, Johnston J, Hannigan MP (2018). Assessing a low-cost methane sensor quantification system for use in complex rural and urban environments. Atmospheric Measurement Techniques.

[CR4] Considine, E. M., Reid, C. E., Ogletree, M. R., & Dye, T. (2021). Improving accuracy of air pollution exposure measurements: Statistical correction of a municipal low-cost airborne particulate matter sensor network. *Environmental Pollution*, 268(B), 115833. 10.1016/j.envpol.2020.115833 10.1016/j.envpol.2020.115833 33120139

[CR5] Eugster, W., Laundre, J., Eugster, J., & Kling, G. W. (2020). Long-term reliability of the Figaro TGS 2600 solid-state methane sensor under low-Arctic conditions at Toolik Lake, Alaska. *Atmospheric Measurement Techniques*, 13(5), 2681–2695, 2020. 10.5194/amt-13-2681-2020

[CR6] Eugster W, Kling GW (2012). Performance of a low-cost methane sensor for ambient concentration measurements in preliminary studies. Atmospheric Measurement Techniques.

[CR7] Fakra DAH, Andriatoavina DAS, Razafindralambo NAMN, Amarillis KA, Andriamampianina JMM (2020). A simple and low-cost integrative sensor system for methane and hydrogen measurement. Sensors International.

[CR8] Gibergans-Baguena, J., Hervada-Sala, C., & Jarauta-Bragulat, E. (2020). The quality of urban air in Barcelona: A new approach applying compositional data analysis methods. *Emerging Science Journal*, 4(2), 113–121. 10.28991/esj-2020-01215

[CR9] Hamzah MAF, Jahim JM, Abdul PM, Asis AJ (2019). Investigation of temperature effect on start-up operation from anaerobic digestion of acidified palm oil mill effluent. Energies.

[CR10] Honeycutt WT, Ley MT, Materer NF (2019). Precision and limits of detection for selected commercially available, low-cost carbon dioxide and methane gas sensors. Sensors.

[CR11] Hu EB, Babcock EL, Bialkowski SE, Jones SB, Tuller M (2014). Methods and techniques for measuring gas emissions from agricultural and animal feeding operations. Critical Reviews in Analytical Chemistry.

[CR12] Hu W, Wan L, Jian Y, Ren C, Jin K, Su X, Bai X, Haick H, Yao M, Wu W (2018). Electronic noses: From advanced materials to sensors aided with data processing. Advanced Materials Technologies.

[CR13] Humidity and temperature sensor datasheet SHT3x-DIS. (2016). Retrieved July 17, 2019, from https://www.mouser.com/datasheet/2/682/Sensirion_Humidity_Sensors_SHT3x_Datasheet_digital-971521.pdf

[CR14] Isaksen ISA, Terje K, Berntsen TK, Dalsøren SB, Eleftheratos K, Orsolini Y, Rognerud B, Stordal F, Søvde OA, Zerefos C, Holmes CD (2014). Atmospheric ozone and methane in a changing climate. Atmosphere.

[CR15] Izumoto, S., Hamamoto, S., Kawamoto, K., Nagamori, M., & Nishimura, T. (2018). Monitoring of methane emission from a landfill site in daily and hourly time scales using an automated gas sampling system. *Environmental Science and Pollution Research, 25*, 24500–24506. 10.1007/s11356-018-2671-1 10.1007/s11356-018-2671-1 30009359

[CR16] Kuula J, Kuuluvainen H, Rönkkö T, Niemi JV, Saukko E, Portin H, Aurela M, Saarikoski S, Rostedt A, Hillamo R, Timonen H (2019). Applicability of optical and diffusion charging-based particulate matter sensors to urban air quality measurements. Aerosol and Air Quality Research.

[CR17] Lee, Y., Yang, P., Chang, C., & Fang, W. (2018). Design and fabrication of MOS type gas sensor with vertically integrated heater using CMOSMEMS technology. *Proceedings*, 2(13), 772. 10.3390/proceedings2130772

[CR18] Lewis, A., Peltier, W. R., & von Schneidemesser, E. (2018). Low-cost sensors for the measurement of atmospheric composition: Overview of topic and future applications*.* Research Report. World Meteorological Organization (WMO), Geneva, Switzerland.

[CR19] MQ-4 sensor technical data. (2019). Retrieved July 17, 2019, from http://image.dfrobot.com/image/data/SEN0129/MQ-4.pdf

[CR20] National Academies of Sciences, Engineering, and Medicine. (2018). *Improving characterization of anthropogenic methane emissions in the United States*. Chapter 3: Methane emission measurement and monitoring methods. Washington, DC: The National Academies Press. 10.17226/24987 30110140

[CR21] Nagahage EAAD, Nagahage ISP, Fujino T (2019). Calibration and validation of a low-cost capacitive moisture sensor to integrate the automated soil moisture monitoring system. Agriculture.

[CR22] Manisalidis I, Stavropoulou E, Stavropoulos A, Bezirtzoglou E (2020). Environmental and health impacts of air pollution: A review. Frontiers in Public Health.

[CR23] Oliver, D. W. (2019). Implications of sampling methods on geospatial mapping of methane sources. Dissertations. West Virginia University. https://researchrepository.wvu.edu/etd/4038

[CR24] Pascal M, Corso M, Chanel O, Declercq C, Badaloni C, Cesaroni G, Henschel S, Meister K, Haluza D, Martin-Olmedo P, Medina S (2013). Assessing the public health impacts of urban air pollution in 25 European cities: Results of the Aphekom project. Science of the Total Environment.

[CR25] Pehme, K. M., Orupõld, K., Kuusemets, V., Tamm, O., Jani, Y., Tamm, T., & Kriipsalu, M. (2020). Field study on the efficiency of a methane degradation layer composed of fine fraction soil from landfill mining. Sustainability, 12, 6209. 10.3390/su12156209

[CR26] Raaschou-Nielsen O, Beelen R, Wang M (2016). Particulate matter air pollution components and risk for lung cancer. Environmental International.

[CR27] Smith KR, Edwards PM, Ivatt PD, Lee JD, Squires F, Dai C, Peltier RE, Evans MJ, Sun Y, Lewis AC (2019). An improved low-power measurement of ambient NO_2_ and O_3_ combining electrochemical sensor clusters and machine learning. Atmospheric Measurement Techniques.

[CR28] Spinelle L, Gerboles M, Kok G, Persijn S, Sauerwald T (2017). Review of portable and low-cost sensors for the ambient air monitoring of benzene and other volatile organic compounds. Sensors.

[CR29] TGS 2611 gas sensor technical data. (2019). TGS 2611 for the detection of methane. Retrieved July 17, 2019, from http://www.figarosensor.com/product/docs/TGS%202611C00(1013).pdf

[CR30] van den Bossche M, Rose NT, De Wekker SFJ (2016). Potential of a low-cost gas sensor for atmospheric methane monitoring. Sensors and Actuators b: Chemical.

[CR31] Wu, F., Lu, Y., Wang, M., Zhang, X., & Yang, C. (2019). Catalytic removal of ozone by Pd/ACFs and optimal design of ozone converter for air purification in aircraft cabin. *Civil Engineering Journal,*5 (8), 1656–1671. 10.28991/cej-2019-03091361

[CR32] Wu S, Ni Y, Li H, Pan L, Yang D, Baccarelli A, Deng F, Chen Y, Shima M, Guo X (2016). Short-term exposure to high ambient air pollution increases airway inflammation and respiratory symptoms in chronic obstructive pulmonary disease patients in Beijing, China. Environment International.

[CR33] Yang S, Liu Y, Wu N, Zhang Y, Svoronos S, Pullammanappallil P (2019). Low-cost, Arduino-based, portable device for measurement of methane composition in biogas. Renewable Energy.

