# Supplementary material for: Assessment of the applicability of a low-cost sensor–based methane monitoring system for continuous multi-channel sampling
Source: Environ Monit Assess. 2021 Jul 23;193(8):509. doi: 10.1007/s10661-021-09290-w (PMC8302541; doi:10.1007/s10661-021-09290-w)
Supplement: Supplementary file 1 — Supplementary file1 (PDF 53 KB) [file 10661_2021_9290_MOESM1_ESM.pdf]

```
;##############################################################################################
;##   I. S. P. Nagahage & E. A. A Dilrukshi
##
;##   2019/02/02
               ##
;## Double Methane sensor Interface                                                         ##
;##############################################################################################

;-----------------------------I2C Device address--------------------------------------------------
I2C_LCD                                 equ         4Eh                        ;slave address for LCD
I2C_ADS1                          equ         92h                        ;slave address for ADS1 module
I2C_ADS2                          equ         90h                        ;slave address for ADS2 module
I2C_SHT                                 equ         88h                        ;slave address for SHT3X

;------------------------------Port declaration------------------------------------------------------
SDA_PIN                           bit   P1.0              ;I2C data line
SCL_PIN                           bit   P1.1              ;I2C clock line
SINK                              bit         P1.2              ;triggering
LED                                     bit         P1.3
LED1                              bit         P1.5

VLV_M                             bit         P0.0              ;valve one
VLV_1                             bit         P0.1
VLV_2                             bit         P0.2
VLV_3                             bit         P0.3
VLV_4                             bit         P0.4
VACUM_PUMP                        bit         P0.5              ;vaccum pump control


;-------------------------------ESP8266 serial communication-------------------------------------------
TimerReload                       equ         0FDh              ;generate 9600boud rate


;-------------------------------RAM data storage buffers--------------------------------------------------

BIT_CNT                                 data  8h                      ; Bank1 register for bit count
BYTE_CNT                          data  9h                      ; Bank1 register for byte count
SLV_ADDR                          data  0Ah                     ; Bank1 register for slave address
ADS_PIN                                 data  0Bh                     ; Select the analog input
ADS_SEL                                 data  0Ch                     ; select ADS module

OUTONES                                 data  0Dh                     ;output of ones colum in 2nd compliment to ascii
```

```
conversion
OUTENS                              data  0Eh                     ;output of 10s
OUTHUND                             data  0Fh                     ;output of 100s
OUTHOUN                             data  10h                     ;output of 1000s
OUTENTH                             data  11h                     ;output of 10000s
SIGNOUT                             data  12h                     ;plus or minus sign



FLAGS                         data  20h                     ;location for bit flags in bit addresable area
NO_ACK                              bit         FLAGS.0                 ;I2C no acknowledge flag
BUS_FAULT                     bit         FLAGS.1                 ;I2C bus fault flag
I2C_BUSY                      bit         FLAGS.2                 ;I2C busy flag
SerialComp                    bit         FLAGS.3                 ;serial complete
Methn_Anlys                   bit         FLAGS.4                 ;methane analysis
Meth_FINISH                   bit         FLAGS.5                 ;last bottle finished and start from the begining
Meth_Tmp_Hum                  bit         FLAGS.6                 ;methane bottle and temp load
Meth_Tmp_Hum1                 bit         FLAGS.7                 ;methane bottle and temp load 2nd methane sensor

LCD_LATCH                     data  21h                     ;Enable latch for lcd data in bit addresable register

LCD_BUFF                      data  30h                     ;data to be written in buffer
ALT_RCV                             data  31h                     ;BCD data convert and store
XMT_DAT                             data  33h                     ;transmit buffer in general  memomry
RCV_DAT                             data  3Ch                     ;recive buffer// aditionally use for misture level
compare function
I2C_DELAY                     data  45h                     ;reserve last general purpose registers for delay
DlayVar                             data  46h                     ;tempory erroe checking led
TMP_DISP                      data  55h                     ;tempry display memory



;-------------------------------Auxillary RAM data storage buffers-------------------------------------------

ARAN0                         xdata 00h                     ;auxilarry ram starting from 00h, 2 bytes required for 2nd
compliment
ARAN1                         xdata 02h
ARAN2                         xdata 04h
ARAN3                         xdata 06h
ARAN4                         xdata 08h
ARAN5                         xdata 0Ah
ARAN6                         xdata 0Ch
```

```
ARAN7                         xdata 0Eh


MOIST1                              xdata 28h                      ;desired moisture level from web input as 2nd
complimentry 2 bytes
MOIST2                              xdata 2Ah
MOIST3                              xdata 2Ch
MOIST4                              xdata 2Eh

SHT1T                         xdata 30h
SHT2T                         xdata 31h
SHT1R                         xdata 32h
SHT2R                         xdata 33h
;-----------------------------RESET---------------------------------------------------------------------------
      org   0
      ajmp                    I2C_RESET

      org                     23h;
      ajmp                    Serial                              ; jump to serial ISR


;-----------------------------I2C control---------------------------------------------------------------------
SEND_STOP:                                                        ;I2C stop condition to release the bus
      clr                     SDA_PIN                                   ;Get SDA ready for stop
      acall             Release_SCL_HIGH              ;Set clock for stop
      mov                     I2C_DELAY,#3
      acall                   DELAY                               ;Delay
      setb              SDA_PIN                                   ;Send I2C stop
      clr               I2C_BUSY                            ;clear I2C busy status
      ret

SEND_MSG:                                                         ;Buffer @R0=Slvaddr,# of byte to be transffered, Data
bytes
      mov               SLV_ADDR,@R0                        ;Initialize slave address
      inc                     R0                                        ;Next address
      mov               BYTE_CNT,@R0                        ;Initialize BYTE_CNT
      inc               R0                                        ;Next address
      acall                   SEND_DATA                           ;send data
      ret                                                               ;return from subroutine

MASTER_CONTROLLER:
      setb              I2C_BUSY                            ;Indicate that I2C frame is in progress
```

```
	clr		NO_ACK				;clear error status flag
	clr		BUS_FAULT
	jnb		SCL_PIN,FAULT			;Check for bus
	jnb		SDA_PIN,FAULT
	clr		SDA_PIN				;Begin I2C start
	mov		I2C_DELAY,#3
	acall		DELAY				;Delay
	clr		SCL_PIN				;Compleate I2C start
	mov		I2C_DELAY,#3
	acall		DELAY				;Delay
	mov		A,SLV_ADDR			;get slave address
	acall		SEND_BYTE			;send slave address
	ret

	FAULT:
		setb		BUS_FAULT		;set fault status
		ret

SEND_BYTE:						;This subroutine send 1 byte information in A and
verrify ack
	mov		BIT_CNT,#8			;set bit count value
	SB_LOOP:
		rlc		A			;send one data bit
		mov		SDA_PIN,C		;put the data bit on pin
		acall		Release_SCL_HIGH	;Drive scl high
		mov		I2C_DELAY,#3
		acall		DELAY			;Delay

		clr		SCL_PIN			;clear scl
		mov		I2C_DELAY,#3
		acall		DELAY			;Delay
		djnz		BIT_CNT,SB_LOOP		;repeat untill all bits sent

		setb		SDA_PIN			;Relaese data line for acknowledge
		acall		Release_SCL_HIGH	;send clock for ackknowledge
		mov		I2C_DELAY,#4
		acall		DELAY			;delay 4 machine cycles
		jnb		SDA_PIN,SB_EX		;check for valid knowledge bit
		setb		NO_ACK			;set status for no acknowledge

			SB_EX:
			clr		SCL_PIN		;finish acknowledge bit
```

```
            mov                         I2C_DELAY,#3
            acall                       DELAY               ;Delay
            ret

SEND_DATA:                                                  ;transmit multipple data bytes
      acall                 MASTER_CONTROLLER       ;Acquire bus and send slave address
      jb                          NO_ACK,SDEX               ;Check for slave not responding

      SD_LOOP:
            mov                   A,@R0                     ;Get data byte from the buffer
            acall           SEND_BYTE                 ;send next data byte
            inc                   R0                              ;adavance buffer point
            jb                    NO_ACK,SDEX               ;check for slave not responding
            djnz            BYTE_CNT,SD_LOOP        ;all bytes sent?

            SDEX:
            acall       SEND_STOP                     ;Done. send I2C stop
            ret

TRANSFER:                                                   ;This subrountine copies data from EPROM reference by
dptr
      clr                   A

      movc            A,@A+DPTR                         ;moves content of DPTR into A
      mov                   @R1,A                           ;copies A into buffer
      inc                   R1                                    ;next address
      inc                   DPTR                            ;next location
      clr             A                                     ;clear ACC

      movc            A,@A+DPTR                         ;moves content of DPTR into A
      mov                   @R1,A                           ;copies A into buffer
      mov             R0,A                              ;copies A into R0 (# of bytes)
      inc             R1                                      ;next address
      inc             DPTR                              ;next location
      clr             A                                       ;clears A

            NEXT:
            movc              A,@A+DPTR                 ;moves content of DPTR into A
            dec                     R0                              ;decrease #of remaiining bytes
            mov                     @R1,A                   ; copies A into buffer
            inc               R1                                ;next address
            inc                     DPTR                    ;next location
```

```
		clr			A				;clears A
		cjne		R0,#0,NEXT		;compre # of bytes remaing
		ret

RECV_MSG:
	mov			SLV_ADDR,@R1			;moves SLV_ADDR from buffer R0 points to
	inc			R1					;Next buffer location
	mov			BYTE_CNT,@R1			;moves BYTE_CNT value into memory location
	acall		RCV_DATA				;calls receive data subroutine
	ret									;returns from receive msg subroutine

RECV_BYTE:
	mov			BIT_CNT,#8				;set bit count

		RB_LOOP:
		acall		Release_SCL_HIGH	;read one data bit
		mov				I2C_DELAY,#3
		acall			DELAY			;Delay
		mov				C,SDA_PIN		;get data bit from pin
		rlc				A				;rotate bit into result byte
		clr				SCL_PIN			;clear scl pin
		mov				I2C_DELAY,#3
		acall			DELAY			;Delay
		djnz		BIT_CNT,RB_LOOP		; repeat until all bit receive
		push		ACC					;save accumulator
		mov				A,BYTE_CNT		;copies byte count into A
		cjne		A,#1,RB_ACK			;check for last byte of frame
		setb		SDA_PIN				;send no acknowledge on last byte
		sjmp		RB_ACLK				;No ACK onlast byte; jump to RB_ACK

		RB_ACK:
			clr			SDA_PIN				;send acknowledge bit

			RB_ACLK:
				acall Release_SCL_HIGH	;send acknowlwdge clock
				pop		ACC				;restore accumulator
				mov		I2C_DELAY,#3
				acall	DELAY			;Delay
				clr		SCL_PIN			;clear scl pin
				setb	SDA_PIN			;clear acknowledge bit
				mov		I2C_DELAY,#4
				acall	DELAY			;Delay
```

```
                        ret                                     ;Return from RECV_BYTE


RCV_DATA:
      inc                             SLV_ADDR                ;set for READ of slave
      acall                     MASTER_CONTROLLER ;acquire bus and send slave address
      jb                              NO_ACK,RDEX             ;check for slave not responding

            RDLoop:
                  acall           RECV_BYTE               ;receive next data byte
                  mov                   @R0,A                   ;save data byte in buffer
                  inc                   R0                            ;advance buffer point
                  djnz            BYTE_CNT,RDLoop         ;Repeat untill all bytes received

                  RDEX:
                        acall SEND_STOP                 ;done, send an I2C stop
                        ret                                   ;Return from RCV_DATA subroutine

Release_SCL_HIGH:
       setb                     SCL_PIN
       jnb                      SCL_PIN,$
       ret

DELAY:
       djnz                     I2C_DELAY,DELAY         ;delay for I2C bus
       ret

;*************************************LCD in I2C bus *********************************************


;-----------------------------upper nibble conversion---------------------------------------------

WRITE_2_NIBBLES:
      mov                             A,LCD_BUFF
      orl                             LCD_LATCH,#0F0h         ;check the value of upper nibble
      orl                             A,#0Fh                  ;Don't affect bits 0-3
      anl                             LCD_LATCH,A             ;High nibble to display
      acall                     LCD_LATCH_E             ;Latching the data
      mov                             A,LCD_BUFF              ;mask lower nibble to write
    swap                        A                       ;second nibble
      orl                             LCD_LATCH,#0F0h         ;Bits 4...7 <- 1
      orl                             A,#0Fh                  ;Don't affect bits 0...3
```

```
	anl				LCD_LATCH,A		;Low nibble to display
	acall			LCD_LATCH_E		;Latching the data
	ret

;----------------------------------LCD Initialization------------------------------------------------
INIT_LCD:
	mov				SLV_ADDR,#I2C_LCD ;lcd slave address loaded
	acall			MASTER_CONTROLLER
	mov				LCD_LATCH,#28h		;RS clear for commnd,R/W clear for write,Clear enable
	acall			LCD_LATCH_E		;Latching the data
	mov				LCD_BUFF,#28h		;LCD intializing command
	acall			WRITE_2_NIBBLES
	mov				LCD_BUFF,#0Ch		;Dispaly on and cursor off
	acall			WRITE_2_NIBBLES
	mov				LCD_BUFF,#06h		;Increment cursor
	acall			WRITE_2_NIBBLES
	acall			INTRO
	acall			SEND_STOP
	ret

;----------------------------------LCD Latch enable disable routing----------------------------------
LCD_LATCH_E:
	setb			LCD_LATCH.2
	mov				A,LCD_LATCH
	acall			SEND_BYTE		;should be edited*******
	clr				LCD_LATCH.2
	mov				A,LCD_LATCH
	acall			SEND_BYTE
	ret

;----------------------------------LCD Clear---------------------------------------------------------
CLEAR_LCD:
	mov				LCD_BUFF,#01h		;clear lcd screen
	acall			WRITE_COMMAND		;
	acall			LCDDelay
	ret

;----------------------------------LCD Data write-----------------------------------------------------
WRITE_TEXT:
	setb			LCD_LATCH.0		;RS set bit for data
	acall			WRITE_2_NIBBLES		;
	ret
```

```
;-------------------------------LCD Command write----------------------------------------------
WRITE_COMMAND:
      clr                               LCD_LATCH.0             ;RS set for command
      acall                       WRITE_2_NIBBLES         ;
      ret


;*************************************ADS in I2C bus *******************************************
ADS_CONFIG:
      mov                               R3,#3
      mov                               R4,#4
      mov                               ADS_SEL,#I2C_ADS1
ADC:
      mov                               XMT_DAT,ADS_SEL         ;load ads2 module address
      ajmp                        AN0


                        AN0:
                              cjne                        R3,#3,AN1
                              mov                               ADS_PIN,#11000011b            ;single shot with dissable
comparator, A0
                              acall                       ADS
                              dec                               R3
                              ajmp                        ADC
                              ret

                        AN1:
                              cjne                        R3,#2,AN2
                              mov                               ADS_PIN,#11010011b            ;single shot with dissable
comparator, A1
                              acall                       ADS
                              dec                               R3
                              ajmp                        ADC
                              ret

                        AN2:
                              cjne                        R3,#1,AN3
                              mov                               ADS_PIN,#11100011b            ;single shot with dissable
comparator, A2
                              acall                       ADS
                              dec                               R3
```

```
                        ajmp                            ADC
                        ret

                  AN3:
                        mov                                    ADS_PIN,#11110011b          ;single shot with dissable
comparator, A3
                        acall                           ADS
                        ret


ADS:
      mov                                   XMT_DAT,ADS_SEL               ;ADS module address load
      mov                                   XMT_DAT+1,#3h                 ;Write address,address pointer register and then
configuration register
      mov                                   XMT_DAT+2,#00000001b    ;select configration register
      mov                                   XMT_DAT+3,ADS_PIN       ;single shot with dissable comparator and select Analog
input
      mov                                   XMT_DAT+4,#10000011b    ;after setting the lower byte of configuration register
      acall                           CONFIGSEND
            CONREADY:
                        mov                         XMT_DAT+1,#2h
                        mov                         R0,#RCV_DAT
                        mov                         R1,#XMT_DAT
                        acall               RECV_MSG
                        acall               SEND_STOP
                        mov                         A,RCV_DAT
                        clr                         C
                        rlc                         A
                        jnc                         CONREADY
                        clr                         C

      mov                                   XMT_DAT+1,#1h                 ;Write address,address pointer register and then
configuration register
      mov                                   XMT_DAT+2,#00000000b    ;select conversion register
      acall                           CONFIGSEND
      mov                                   XMT_DAT+1,#2h
      mov                                   R0,#RCV_DAT
      mov                                   R1,#XMT_DAT
      acall                           RECV_MSG
      acall                           SEND_STOP
      acall                           AARAN0
      ret
```

```
CONFIGSEND:
      mov                               R0,#XMT_DAT
      acall                       SEND_MSG
      acall                       SEND_STOP
      mov                               R7,#1h
      ret


SECOMPL:
      ;mov                              dptr,#ARAN0
      movx                        A,@dptr
      mov                               RCV_DAT,A
      inc                               dptr
      movx                        A,@dptr
      mov                               RCV_DAT+1,A
      mov                               A,RCV_DAT               ;
      rlc                               A
      jc                                NEG                           ;if carry is there, it shpild be a negative
number
      mov                               SIGNOUT,#"+"            ;ascii pluss
      ajmp                        BIN2BCD
      ret

                        NEG:
                              mov               SIGNOUT,#"-" ;ascii minus
                              clr               C                 ;clear the carry
                              mov               A,RCV_DAT+1  ;move lower byte
                              cpl               A                 ;compliment it
                              add               A,#1         ;add one
                              mov               RCV_DAT+1,A  ;result store in RCV_DATA+1 memory space
                              mov         A,RCV_DAT    ;
                              cpl               A
                              addc        A,#0
                              mov               RCV_DAT,A

BIN2BCD:
      mov                               OUTONES,#00             ;clear memomry
      mov                               OUTENS,#00
      mov                               OUTHUND,#00
      mov                               OUTHOUN,#00
      mov                               OUTENTH,#00
```

```
        mov         B,#10
        mov         A,RCV_DAT+1         ;get low data byte
        div         AB                        ;divide it by 10
        mov         OUTONES,B           ;save the remainder in 1s colum
        mov         B,#10
        div         AB                        ;divide quateint by 10
        mov         OUTENS,B            ;save the remainder
        mov         OUTHUND,A           ;save the last dgit
        mov         A,RCV_DAT           ;get the higher byte
        cjne     A,#00,HIBYT       ;
        ajmp     ASCIWRITE         ;LCD display

HIBYT:
        mov         A,#6                ;if there is a value in higer byte decerse it and add
        add         A,OUTONES           ;256 to corresponding digits
        mov         B,#10
        div         AB
        mov         OUTONES,B           ;save the 1s colom after adding 6
        add         A,#5
        add         A,OUTENS
        mov         B,#10
        div         AB
        mov         OUTENS,B            ;save the remainder after adding 5
        add         A,#2
        add         A,OUTHUND
        mov         B,#10               ;devide by 10
        div         AB
        mov         OUTHUND,B           ;save the remainder after adding 2
        add         A,OUTHOUN
        mov         OUTHOUN,A
        djnz     RCV_DAT,HIBYT     ;decrease and do the process if highr byte is still not zero
        mov         B,#10                     ;if zero, stop the loop and add to 1000 clom and 10000
colom
        mov         A,OUTHOUN
        div         AB
        mov         OUTHOUN,B
        mov         OUTENTH,A

ASCIWRITE:
        mov         A,OUTONES
        add         A,#30h
```

```
        mov                 OUTONES,A
        mov                 A,OUTENS
        add                 A,#30h
        mov                 OUTENS,A
        mov                 A,OUTHUND
        add                 A,#30h
        mov                 OUTHUND,A
        mov                 A,OUTHOUN
        add                 A,#30h
        mov                 OUTHOUN,A
        mov                 A,OUTENTH
        add                 A,#30h
        mov                 OUTENTH,A
        acall           SOIL_TMP_HUM
        mov                 SLV_ADDR,#I2C_LCD ;lcd slave address loaded
        acall           MASTER_CONTROLLER
        mov                 LCD_BUFF,#0C0h
        acall           WRITE_COMMAND
        mov                 LCD_BUFF,SIGNOUT
        acall           WRITE_TEXT
        mov                 LCD_BUFF,OUTENTH
        acall           WRITE_TEXT
        mov                 LCD_BUFF,OUTHOUN
        acall           WRITE_TEXT
        mov                 LCD_BUFF,OUTHUND
        acall           WRITE_TEXT
        mov                 LCD_BUFF,OUTENS
        acall           WRITE_TEXT
        mov                 LCD_BUFF,OUTONES
        acall           WRITE_TEXT
        acall           SEND_STOP
        ret
;*************************************Auxilary ram for temporarary data storage ***********************
AARAN0:
        cjne            R4,#4h,AARAN1
        mov                 dptr,#ARAN0
        dec                 R4
        ajmp            datamov
        ret

AARAN1:
        cjne            R4,#3h,AARAN2
```

```
	mov		dptr,#ARAN1
	dec		R4
	ajmp	datamov
	ret

AARAN2:
	cjne	R4,#2h,AARAN3
	mov		dptr,#ARAN2
	dec		R4
	ajmp	datamov
	ret

AARAN3:
	cjne	R4,#1h,datamovExit
	mov		dptr,#ARAN3
	dec		R4
datamov:
	mov		A,RCV_DAT
	movx	@dptr,A
	inc		dptr
	mov		A,RCV_DAT+1
	movx	@dptr,A
	ret
datamovExit:
	ajmp check
	ret


;***************************************SHT3X Temp & Humidity ******************************************

SHT_INIT:
	mov		XMT_DAT,#I2C_SHT	;SHT module address load
	mov		XMT_DAT+1,#2h		;have to send two_byte single shot command
	mov		XMT_DAT+2,#2Ch		;MSB
	mov		XMT_DAT+3,#6h		;single shot with dissable comparator and select
Analog input
	mov		R0,#XMT_DAT
	acall	SEND_MSG
	acall	SEND_STOP
	mov		XMT_DAT+1,#5h
	mov		R0,#RCV_DAT
	mov		R1,#XMT_DAT
```

```
	acall		RECV_MSG
	acall		SEND_STOP
	mov			dptr,#SHT1T
	mov			A,RCV_DAT
	movx		@dptr,A
	inc			dptr
	mov			A,RCV_DAT+1
	movx		@dptr,A
	inc			dptr
	mov			A,RCV_DAT+3
	movx		@dptr,A
	inc			dptr
	mov			A,RCV_DAT+4
	movx		@dptr,A


	mov			dptr,#SHT1T
	movx		A,@dptr
	mov			RCV_DAT,A
	inc			dptr
	movx		A,@dptr
	mov			RCV_DAT+1,A
	mov			R7,#2h
	mov			SIGNOUT,#"0" ; to avoid unneccery - sign from ads inputs
	acall		BIN2BCD
	acall		ESP8266
	acall		LED2
	mov			dptr,#SHT1R
	movx		A,@dptr
	mov			RCV_DAT,A
	inc			dptr
	movx		A,@dptr
	mov			RCV_DAT+1,A
	mov			R7,#3h
	acall		BIN2BCD
	acall		ESP8266
	acall		LED2

	ret

;*************************************ESP8266 input ****************************************
```

```
ESP8266:
      mov                            IE, #90h                ;10010000-enable serial intrupt
      mov                                  DPTR,#smsg1             ;
      acall                          SBUFLOAD
      mov                                  R0,#ALT_RCV
      acall                          CRLF

SerialR:
      jnb                            RI,$
      clr                                  RI
      acall                          SINKCHK
      ajmp                           ATCWJAP
      ret

Serial:
      reti



SBUFLOAD:
      clr                                  TI
      clr                                  A                       ;clear Accumulator for any previous data
      movc                                 A,@A+DPTR               ;load the first character in accumulator
      jz                             sexit                   ;go to exit if zero
      mov                                  SBUF,A
      jnb                                  TI,$        ;
      inc                                  DPTR                    ;increment data pointer
      sjmp                                 SBUFLOAD          ;jump back to send the next character

            sexit:
                  ret                                                  ;End of routine

ATCWJAP:
      mov                                  DPTR,#smsg2             ;
      acall                          SBUFLOAD
      acall                          COMASUB
      mov                                  DPTR,#smsg3
      acall                          SBUFLOAD
      acall                          COMASUB
      acall                          COMASUB1
      acall                          COMASUB
      mov                                  DPTR,#smsg4
```

```
		acall			SBUFLOAD
		acall			COMASUB
		mov				R0,#ALT_RCV
		acall			CRLF
		acall			SINKCHK
		ATCWJAPR:
				jnb				RI,$
				clr					RI
				;mov					R0,#ALT_RCV
				;acall					CRLF
				;acall					SINKCHK
				ajmp				ATCIPSTRT
				ret

CRLF:
		mov				SBUF,#0Dh
		jnb				TI,$
		clr				TI
		mov				SBUF,#0Ah
		jnb				TI,$
		clr				TI
		ret

ATCIPSTRT:
		mov					R0,#ALT_RCV
		mov					DPTR,#smsg5		;
		acall			SBUFLOAD
		acall			COMASUB
		mov					DPTR,#smsg6
		acall			SBUFLOAD
		acall			COMASUB
		acall			COMASUB1
		acall			COMASUB
		mov					DPTR,#smsg7
		acall			SBUFLOAD
		acall			COMASUB
		acall			COMASUB1
		mov					DPTR,#smsg8
		acall			SBUFLOAD
		mov					R0,#ALT_RCV
		acall			CRLF
		acall			SINKCHK
```

```
    ATCIPSTRTR:
            jnb                         RI,$
            clr                             RI
            ajmp                        ATCIPSEND
            ret
ATCIPSEND:
    mov                         R0,#ALT_RCV
    mov                         DPTR,#smsg9
    acall                   SBUFLOAD
    acall                   CRLF
    jnb                         RI,$
    clr                         RI
    acall                   SELECTR
    mov                         SBUF,SIGNOUT
    jnb                         TI,$
    clr                         TI
    mov                         SBUF,OUTENTH
    jnb                         TI,$
    clr                         TI
    mov                         SBUF,OUTHOUN
    jnb                         TI,$
    clr                         TI
    mov                         SBUF,OUTHUND
    jnb                         TI,$
    clr                         TI
    mov                         SBUF,OUTENS
    jnb                         TI,$
    clr                         TI
    mov                         SBUF,OUTONES
    jnb                         TI,$
    clr                         TI
    mov                         SBUF,#' '
    jnb                         TI,$
    clr                         TI
    mov                         R0,#ALT_RCV
    acall                   CRLF
    acall                   CRLF
    clr                         RI
    ATCIPSENDR:
            mov                             DPTR,#smsg13
            acall                       SBUFLOAD
            acall                       CRLF
```

```
                  jnb                            RI,$
                  clr                                  RI
                  ret

SELECTR:
      jnb                            Methn_Anlys,SELECTRM
 SL_MINUS1:
      cjne                     R5,#3,SL_MINUS2
      jb                             Meth_Tmp_Hum,Methane_2L1
      mov                            DPTR,#smsg14
      setb                     Meth_Tmp_Hum
      dec                            R6
      acall                    SBUFLOAD
      ret

      Methane_2L1:
            jb                             Meth_Tmp_Hum1,SELECTR1
            mov                            DPTR,#smsg18
            setb                     Meth_Tmp_Hum1
            dec                            R6
            acall                    SBUFLOAD
            ret

 SL_MINUS2:
      cjne                     R5,#2,SL_MINUS3
      jb                             Meth_Tmp_Hum,Methane_2L2
      mov                            DPTR,#smsg15
      setb                     Meth_Tmp_Hum
      dec                            R6
      acall                    SBUFLOAD
      ret

      Methane_2L2:
            jb                             Meth_Tmp_Hum1,SELECTR1
            mov                            DPTR,#smsg19
            setb                     Meth_Tmp_Hum1
            dec                            R6
            acall                    SBUFLOAD
            ret

 SL_MINUS3:
      cjne                     R5,#1,SL_MINUS4
```

```
    jb                          Meth_Tmp_Hum,Methane_2L3
    mov                         DPTR,#smsg16
    setb                    Meth_Tmp_Hum
    dec                         R6
    acall                   SBUFLOAD
    ret

    Methane_2L3:
        jb                          Meth_Tmp_Hum1,SELECTR1
        mov                         DPTR,#smsg20
        setb                    Meth_Tmp_Hum1
        dec                         R6
        acall                   SBUFLOAD
        ret

SL_MINUS4:
    cjne                    R5,#0,SELECTRM
    jb                          Meth_Tmp_Hum,Methane_2L4
    mov                         DPTR,#smsg17
    setb                    Meth_Tmp_Hum
    dec                         R6
    acall                   SBUFLOAD
    ret

        Methane_2L4:
        jb                          Meth_Tmp_Hum1,SELECTR1
        mov                         DPTR,#smsg21
        setb                    Meth_Tmp_Hum1
        dec                         R6
        acall                   SBUFLOAD
        ret



    SELECTRM:
    cjne                    R6,#3,SELECTRM2
    mov                         DPTR,#smsg10
    acall                   SBUFLOAD
    dec                         R6
    ret

    SELECTRM2:
```

```
	cjne			R6,#2,SELECTR1
	mov				DPTR,#smsg23
	acall			SBUFLOAD
	dec				R6
	ret

	SELECTR1:
	cjne			R6,#1,SELECTR2
	mov				DPTR,#smsg11
	acall			SBUFLOAD
	dec				R6
	ret

		SELECTR2:
		mov				DPTR,#smsg12
		acall			SBUFLOAD
		clr				Meth_Tmp_Hum
		clr				Meth_Tmp_Hum1
		ret



COMASUB:
	mov				SBUF,#'"'
	jnb				TI,$
	clr				TI
	ret

COMASUB1:
	mov				SBUF,#','
	jnb				TI,$
	clr				TI
	ret

;******************Methane _mesurement*****************************************************************

methane_mesure:

	cjne			R5,#4h,CHK_BOTTLE_2

CHK_BOTTLE_1:
	mov				DlayVar+7,#8h
```

```
	acall			MTHNE_EMTY
	clr				VLV_M
	clr				VLV_1
	clr				VACUM_PUMP
	acall			Motor100ms
	;acall				Delay1
	;acall				Delay1
	setb			VACUM_PUMP
	setb			VLV_1
	ajmp			MTHNE_VLU_UPLOAD
	ret

CHK_BOTTLE_2:
	cjne			R5,#3h,CHK_BOTTLE_3
	mov				DlayVar+7,#8h
	acall			MTHNE_EMTY
	clr				VLV_M
	clr				VLV_2
	clr				VACUM_PUMP
	acall			Motor100ms
	;acall				Delay1
	;acall				Delay1
	setb			VACUM_PUMP
	setb			VLV_2
	ajmp			MTHNE_VLU_UPLOAD
	ret

CHK_BOTTLE_3:
	cjne			R5,#2h,CHK_BOTTLE_4
	mov				DlayVar+7,#8h
	acall			MTHNE_EMTY
	clr				VLV_M
	clr				VLV_3
	clr				VACUM_PUMP
	acall			Motor100ms
	;acall				Delay1
	;acall				Delay1
	setb			VACUM_PUMP
	setb			VLV_3
	ajmp			MTHNE_VLU_UPLOAD
	ret
```

```
CHK_BOTTLE_4:
	mov		DlayVar+7,#8h
	acall		MTHNE_EMTY
	clr		VLV_M
	clr		VLV_4
	clr		VACUM_PUMP
	acall		Motor100ms
	;acall		Delay1
	;acall		Delay1
	setb		VACUM_PUMP
	setb		VLV_4
	setb		Meth_FINISH

MTHNE_VLU_UPLOAD:
	dec		R5
	mov		IE,#00h			;00000000-disable all interupts serial intrupt
	ajmp		ADS_set
	ret

MTHNE_EMTY:
	clr		VLV_M
	clr		VACUM_PUMP
	acall		Motor100ms
	;acall		Delay1
	setb		VLV_M
	acall		Delay1
	acall		Delay1
	acall		Delay1
	acall		Delay1
	acall		Delay1
	acall		Delay1
	acall		Delay1
	acall		Delay1
	acall		Delay1
	acall		Delay1
	acall		Delay1
	acall		Delay1
	acall		Delay1
	acall		Delay1
	acall		Delay1
	acall		Delay1
	acall		Delay1
```

```
	acall				Delay1
	acall				Delay1
	acall				Delay1
	acall				Delay1
	acall				Delay1
	acall				Delay1
	acall				Delay1
	setb				VACUM_PUMP
	ret

Motor100ms:
	mov	DlayVar,#100
	mov	DlayVar+1,#80
	mov	DlayVar+2,#2
	acall DlayLoop
	ret

;___________________MAIN PROGRAM________________________________________________________________________

I2C_RESET:
	mov					SP,#56H				;set stack to general porpose register 57H
	acall				INIT_LCD			;lcd initialization
	mov				TMOD,#20h			;timer1, mode 2(auto-reload)
	mov				TH1, #0FDh			;9600 baud rate
	mov				SCON,#50h			;8-bit, 1 stop, REN enable
	setb				TR1					;starts timer1

hr_dlay:
	clr					Methn_Anlys			;flag clear for normal measurements
	clr					Meth_FINISH
	acall				MTHNE_EMTY
	mov	DlayVar+5,#144						;make 6 hrs delay (DlayVar+5,#150, DlayVar+6,#2)
	mov DlayVar+6,#7						;make one day delay DlayVar+5,#144,mov DlayVar+6,#7

firt_round:
	djnz				DlayVar+5,ADS_set ;
	djnz				DlayVar+6,rload		;again relaod value to DlayVar+5
	setb				Methn_Anlys			;start methane analysis
	mov					R5,#4h				;check 4 bottles
MJ: ajmp				methane_mesure		;
	ret
```

```
rload:
      mov                                   DlayVar+5,#144          ;again relaod value to DlayVar+5
      ajmp                            firt_round
      ret

ADS_set:
      mov                                   R6,#3h
      djnz                            DlayVar+7,AD
      jb                                    Meth_FINISH,hr_dlay
      jb                                    Methn_Anlys,MJ
AD:
      mov                             IE, #00h                ;00000000-disable all interupts serial intrupt
      acall                           ADS_CONFIG

check:
      mov                                   R7,#01h
      mov                                   dptr,#ARAN0             ;methane sensor 1 reading conversion
      acall                           SECOMPL
      acall                           ESP8266

      mov                                   R7,#04h
      mov                                   dptr,#ARAN2             ;methane sensor 2 reading conversion
      acall                           SECOMPL
      acall                           ESP8266


      acall                           SHT_INIT


LED11:
      setb                            LED
      acall                                 Delay1
      cpl                                   LED
      acall                           Delay1
      mov                                   R6,#3h
      ajmp                            firt_round
      ret

SINKCHK:
      setb                            SINK
      acall                                 Delay1
      cpl                                   SINK
```

```
        acall                         Delay1
        ret


LED2:
        setb                          LED1
        acall                               Delay1
        cpl                                 LED1
        acall                         Delay1
        ret


Delay1:                                                                     ;1-second dlay (1666666 cycles)
        mov   DlayVar,#250
        mov   DlayVar+1,#250
        mov   DlayVar+2,#5
DlayLoop:
        djnz  DlayVar,DlayLoop
        djnz  DlayVar+1,DlayLoop
        djnz  DlayVar+2,DlayLoop
        ret


LCDDelay:
        mov         DlayVar+3,#255
        mov         DlayVar+4,#3
        DlayLoop1:
        djnz  DlayVar+3,DlayLoop1
        djnz  DlayVar+4,DlayLoop1
        ret

DISPLAY:
        clr                                 A                       ;clear Accumulator for any previous data
        movc                                A,@A+DPTR               ;load the first character in accumulator
        jz                            exit              ;go to exit if zero
        mov                                 LCD_BUFF,A
        acall                               WRITE_TEXT              ;send first char
        inc                                 DPTR                    ;increment data pointer
        sjmp                                DISPLAY                 ;jump back to send the next character

              exit:
                    ret                                                                 ;End of routine
```

```
INTRO:
      mov                          LCD_BUFF,#081h          ;
      acall                              WRITE_COMMAND           ;
      mov                                DPTR,#msg1              ;
      acall                              DISPLAY                       ;
      mov                          LCD_BUFF,#0C0h          ;
      acall                              WRITE_COMMAND           ;
      mov                          DPTR,#msg2              ;
      acall                              DISPLAY                       ;
      acall                        SEND_STOP
      ret

INITIALPOSSITIONING:
      mov                                SLV_ADDR,#I2C_LCD ;lcd slave address loaded
      acall                        MASTER_CONTROLLER
      acall                        CLEAR_LCD
      mov                          LCD_BUFF,#080h          ;
      acall                              WRITE_COMMAND           ;
      mov                                DPTR,#msg9              ;
      acall                              DISPLAY                       ;
      mov                          LCD_BUFF,#0C0h          ;
      acall                              WRITE_COMMAND           ;
      mov                          DPTR,#msg10             ;
      acall                              DISPLAY                       ;
      acall                        SEND_STOP
      ret

SOIL_TMP_HUM:
      mov                                SLV_ADDR,#I2C_LCD ;lcd slave address loaded
      acall                        MASTER_CONTROLLER
      acall                        CLEAR_LCD
      mov                          LCD_BUFF,#080h          ;
      acall                              WRITE_COMMAND           ;

      SOIL:
      cjne                         R7,#01h,MET2
      mov                                DPTR,#msg20             ;
      acall                              DISPLAY                       ;
      acall                        SEND_STOP
      ret
```

```
MET2:
cjne                         R7,#04h,TMP
mov                                DPTR,#msg23            ;
acall                              DISPLAY                      ;
acall                        SEND_STOP
ret

TMP:
cjne                         R7,#02h,HUM
mov                                DPTR,#msg21            ;
acall                              DISPLAY                      ;
acall                        SEND_STOP
ret

HUM:
mov                          R7,#00h
mov                                DPTR,#msg22            ;
acall                              DISPLAY                      ;
acall                        SEND_STOP
ret



INITIALPOSSITIONINGDETECTED:
mov                                SLV_ADDR,#I2C_LCD ;lcd slave address loaded
acall                        MASTER_CONTROLLER
acall                        CLEAR_LCD
mov                          LCD_BUFF,#080h         ;
acall                              WRITE_COMMAND          ;
mov                                DPTR,#msg11            ;
acall                              DISPLAY                      ;
mov                          LCD_BUFF,#0C0h         ;
acall                              WRITE_COMMAND          ;
mov                          DPTR,#msg12            ;
acall                              DISPLAY                      ;
acall                        SEND_STOP
ret

Error:
mov                                SLV_ADDR,#I2C_LCD ;lcd slave address loaded
acall                        MASTER_CONTROLLER
```

```
        mov                         LCD_BUFF,#084h          ;
        acall                             WRITE_COMMAND           ;
        mov                               DPTR,#msg3              ;
        acall                             DISPLAY                       ;
        mov                         LCD_BUFF,#0C0h          ;
        acall                             WRITE_COMMAND           ;
        mov                         DPTR,#msg4              ;
        acall                             DISPLAY                       ;
        acall                       SEND_STOP
        ret

Scan:
        mov                               SLV_ADDR,#I2C_LCD ;lcd slave address loaded
        acall                       MASTER_CONTROLLER
        acall                       CLEAR_LCD
        mov                         LCD_BUFF,#080h          ;
        acall                             WRITE_COMMAND           ;
        mov                               DPTR,#msg5              ;
        acall                             DISPLAY                       ;
        mov                         LCD_BUFF,#0C0h          ;
        acall                             WRITE_COMMAND           ;
        mov                         DPTR,#msg6              ;
        acall                             DISPLAY                       ;
        acall                       SEND_STOP
        ret

PLATE_CORDING_DISPLAY:
        mov                               SLV_ADDR,#I2C_LCD ;lcd slave address loaded
        acall                       MASTER_CONTROLLER
        acall                       CLEAR_LCD
        mov                         LCD_BUFF,#080h          ;
        acall                             WRITE_COMMAND           ;
        mov                               DPTR,#msg13             ;
        acall                             DISPLAY                       ;
        mov                         LCD_BUFF,#085h          ;
        acall                             WRITE_COMMAND           ;
        mov                         LCD_BUFF,TMP_DISP
        acall                             WRITE_TEXT              ;
        mov                         LCD_BUFF,#0C0h          ;
        acall                             WRITE_COMMAND           ;
        mov                         DPTR,#msg14             ;
        acall                             DISPLAY                       ;
```

```
        acall                   SEND_STOP
        ret


ANDIGIT:
        mov                             SLV_ADDR,#I2C_LCD ;lcd slave address loaded
        acall                   MASTER_CONTROLLER
        acall                   CLEAR_LCD
        mov                     LCD_BUFF,#080h          ;
        acall                           WRITE_COMMAND           ;
        mov                             DPTR,#msg7              ;
        acall                           DISPLAY                         ;
        mov                     LCD_BUFF,#0C0h          ;
        acall                           WRITE_COMMAND           ;
        mov                     DPTR,#msg8              ;
        acall                           DISPLAY                         ;
        acall                   SEND_STOP
        ret



CLK_SETUP:
        mov                             SLV_ADDR,#I2C_LCD ;lcd slave address loaded
        acall                   MASTER_CONTROLLER
        acall                   CLEAR_LCD
        mov                     LCD_BUFF,#080h          ;
        acall                           WRITE_COMMAND           ;
        mov                             DPTR,#msg               ;
        acall                           DISPLAY                         ;
        mov                     LCD_BUFF,#0C0h          ;
        acall                           WRITE_COMMAND           ;
        mov                     DPTR,#msgm              ;
        acall                           DISPLAY                         ;
        acall                   SEND_STOP
        ret

msg1: db "Methane Monitr",00h;
msg2: db "Systm Initial...",00h;

msg3: db "Error.",00h;
msg4: db "I2C bus fault...",00h;
```

```
msg5: db "Scanning plates-",00h;
msg6: db "Plz don't shake",00h;

msg7: db "Plate detected",00h;
msg8: db " ",00h;

msg9: db "Initial position",00h;
msg10:db "Motor turn left",00h;

msg11:db "Reached end",00h;
msg12:db "Motor Stopped",00h;

msg13:db "PLATE  DETECTED",00h;
msg14:db "Assignd Cordintes",00h;

msg15:db "  Plates Loaded",00h;
msg16:db "Motor turn intil",00h;

msg20:db "Methane Sensor 1: ",00h;

msg21:db "Temperature: ",00h;

msg22:db "Air Humidity: ",00h;

msg23:db "Methane Sensor 2: ",00h;

msg: db ":  :       /",00h;
msgm: db "System standby",00h;


;***********************************Serial ESP commands**********************

smsg1: db "AT",00h;          AT command expecting OK from module
smsg2: db "AT+CWJAP=",00h
smsg3: db "106F3F75645A",00h
smsg4: db "nkb5ah43e4gr8",00h
smsg5: db "AT+CIPSTART=",00h
smsg6: db "TCP",00h
smsg7: db "184.106.153.149",00h
smsg8: db "80",00h
smsg9: db "AT+CIPSEND=55",00h
smsg10: db "GET /update?key=I8IOVSN9OBV65BO0&field1=",00h
```

```
smsg11: db "GET /update?key=I8IOVSN9OBV65BO0&field5=",00h
smsg12: db "GET /update?key=I8IOVSN9OBV65BO0&field6=",00h
smsg23: db "GET /update?key=I8IOVSN9OBV65BO0&field8=",00h

smsg14: db "GET /update?key=I8IOVSN9OBV65BO0&field2=",00h
smsg15: db "GET /update?key=I8IOVSN9OBV65BO0&field3=",00h
smsg16: db "GET /update?key=I8IOVSN9OBV65BO0&field4=",00h
smsg17: db "GET /update?key=I8IOVSN9OBV65BO0&field7=",00h

smsg18: db "GET /update?key=VBUDSJQ4JM3UZ5SU&field1=",00h
smsg19: db "GET /update?key=VBUDSJQ4JM3UZ5SU&field2=",00h
smsg20: db "GET /update?key=VBUDSJQ4JM3UZ5SU&field3=",00h
smsg21: db "GET /update?key=VBUDSJQ4JM3UZ5SU&field4=",00h

smsg13: db "AT+CIPCLOSE",00h

;ayesha fiest floor lab - smsg3: db "Buffalo-G-5474",00h, smsg4: db "8rve5afib6ff6",00h
;kawai sensei 4th floor lab - 0024A5302ECF,j6u8i2ifytwjm
;homw wifi - 106F3F75645A , nkb5ah43e4gr8
;smsg3: db "Kawai-Lab3",00h
;smsg4: db "saiboukinou",00h
end
```
